# Aeromonas hydrophila Induces Skin Disturbance through Mucosal Microbiota Dysbiosis in Striped Catfish (*Pangasianodon hypophthalmus*)

**DOI:** 10.1128/msphere.00194-22

**Published:** 2022-06-29

**Authors:** Li-Hsuan Chen, Chia-Hsuan Lin, Ru-Fang Siao, Liang-Chun Wang

**Affiliations:** a Department of Marine Biotechnology and Resources, National Sun Yet-Sen University, Kaohsiung, Taiwan, Republic of China; National Institute of Advanced Industrial Science and Technology

**Keywords:** *Aeromonas hydrophila*, aquatic infection, host-pathogen interactions, mucosal microbiota, skin immune response

## Abstract

Bacterial pathogens are well equipped to adhere to and initiate infection in teleost fish. Fish skin mucus serves as the first barrier against environmental pathogens. The mucus harbors commensal microbes that impact host physiological and immunological responses. However, how the skin mucosal microbiota responds to the presence of pathogens remains largely unexplored. Thus, little is known about the status of skin mucus prior to infection with noticeable symptoms. In this study, we investigated the interactions between pathogens and the skin mucosal microbiota as well as the fish skin immune responses in the presence of pathogens. Striped catfish (Pangasianodon hypophthalmus) were challenged with different concentrations of the bacterial pathogen Aeromonas hydrophila (AH), and the skin immune response and the mucosal microbiota were examined by quantitative PCR (qPCR) and 16S rRNA gene sequence analysis. We determined that the pathogen concentration needed to stimulate the skin immune response was associated with significant mucosal microbiota changes, and we reconfirmed these observations using an *ex vivo* fish skin model. Further analysis indicated that changes in the microbiota were attributed to a significant increase in opportunistic pathogens over AH. We concluded that the presence and increase of AH result in dysbiosis of the mucosal microbiota that can stimulate skin immune responses. We believe that our work sheds light on host-pathogen-commensal microbiota interactions and therefore contributes to aquaculture fish health.

**IMPORTANCE** The fish skin mucosal microbiota is essential in modulating the host response to the presence of pathogens. Our study provides a platform to study both the correlation and causation of the interactions among the pathogen, fish skin, and the skin mucosal microbiota. Based on these findings, we provide the first mechanistic information on how mucosal microbiota changes induced by the pathogen AH result in skin disturbance with immune stimulation in striped catfish in the natural state and a potential direction for early-infection screening. Thus, this study is highly significant in the prevention of fish disease.

## INTRODUCTION

Fish skin serves as the first line of defense against pathogens and plays vital functions in the immunity, osmoregulation, and endocrine signaling of fish (1). The outer layer of fish skin is the mucus layer secreted by epidermal goblet cells (2). The mucus is vital for the immune system and protects against pathogens by physical, chemical, and biological implements (3–5). The physical and chemical properties allow constant mucus shedding, and the mucus contains host-secreted antimicrobials such as lysozyme, immunoglobulin, and lectins (6, 7). However, mucus also has an interactive and complex role in regulating homeostasis (8–10). The microbiota, defined as the microbial community with distinct physicochemical properties, has evolved to reside with the host and can regulate host-pathogen development (11, 12). The regulation of the microbial community in the mucosal epithelium includes enhancement of the epithelial barrier, development of the immune system, and nutrient acquisition (13–16). In aquaculture, although the mucosal microbiota is important in protecting the host from pathogens by stimulating the immune system (11, 17), the complex interaction of the mucosal microbiota with the pathogens and the fish skin immune system is still unexplored.

Pangasianodon hypophthalmus (striped catfish) is a major freshwater species and dominates 2% of global live-weight aquaculture production (18). Nonetheless, bacterial pathogens can cause production losses (19, 20). The bacterium that commonly causes catfish disease is Aeromonas hydrophila (AH) (20–23). AH is the causative agent of motile *Aeromonas* septicemia (MAS) in farmed striped catfish, especially in the juvenile stage (24). Previous studies have reported the molecular responses of catfish after bacterial infection and described the genomic and genetic bases of disease development (25). Previous studies have also shown that catfish with mucus scraping could successfully initiate AH infection, while undisturbed mucus did not result in infection regardless of any dose of AH (26, 27), hinting at an essential role of the skin mucus in interfering with the entry of pathogens in the natural state. However, the initial interaction between mucus and pathogens along with skin has not been assessed. Therefore, this study aims to mimic the natural state to understand the events in the skin mucus prior to potential infection or damage.

## RESULTS

### Immune stimulation was found in skin tissue in response to AH challenge.

**FIG 1:**
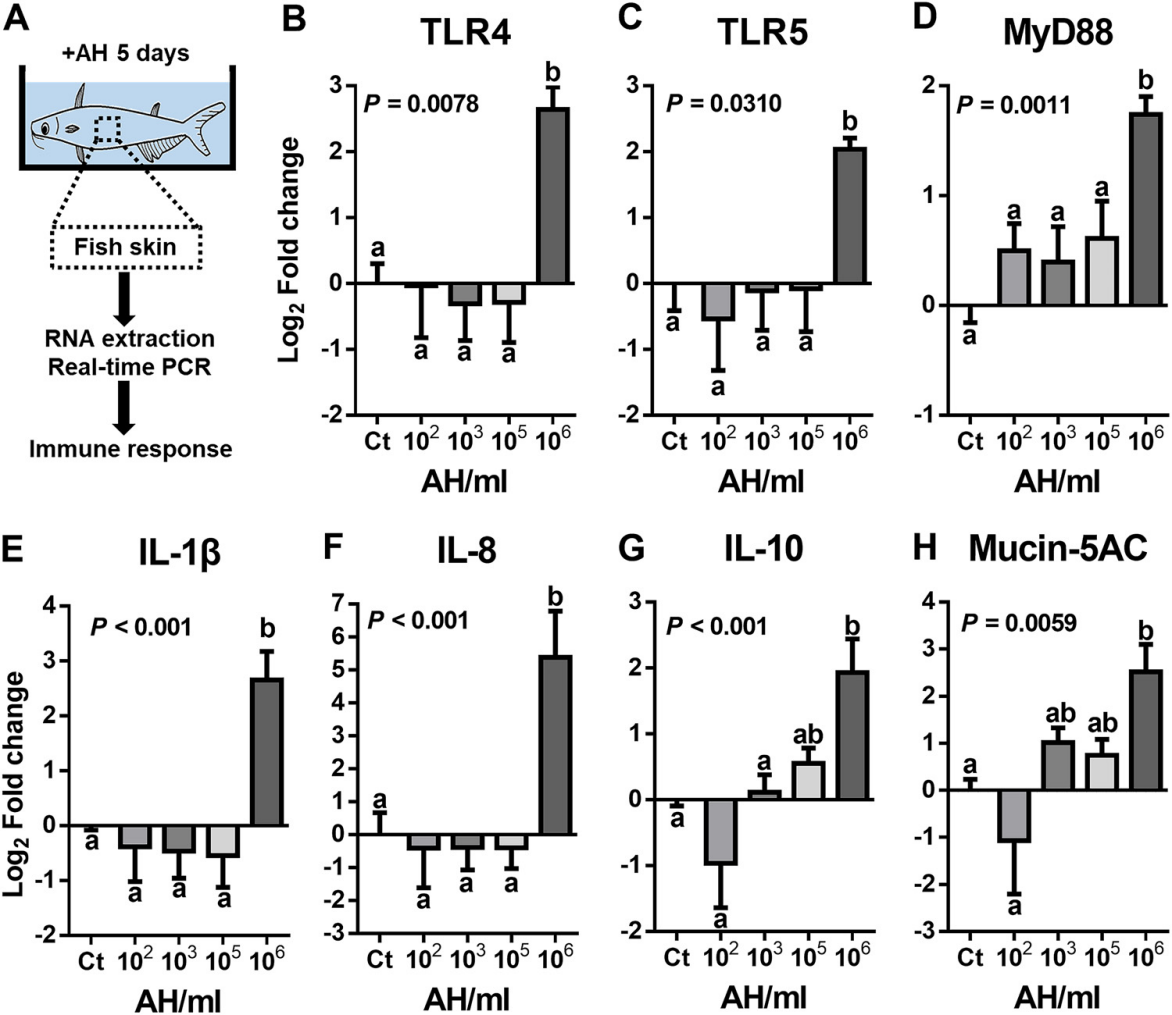
Fish skin immune response after AH challenge. qPCR of AH-challenged and nonchallenged fish skin was performed to quantify the immune response. (A) Illustration of the experiment. (B to H) Expression levels of TLR4 (B), TLR5 (C), MyD88 (D), IL-1β (E), IL-8 (F), IL-10 (G), and mucin-5AC (H) (*n* = 8 fish in each group). Statistical significance was determined by one-way ANOVA followed by Tukey’s *post hoc* test at a *P* value of <0.05 (if treatment groups share the same lowercase letter, then the differences between the groups are not statistically significant).

To validate the host immune response of striped catfish skin under AH challenge, real-time quantitative PCR (qPCR) was used to measure the expression levels of targeted immune genes in the skin (Fig. 1A). Targeted genes were selected based on previous studies and included Toll-like receptors (TLR4 and TLR5), a signal transduction adaptor (MyD88), proinflammatory cytokines (interleukin-1 β [IL-1β] and IL-8), an anti-inflammatory cytokine (IL-10), and mucus secretion (mucin-5AC). We found that 10^6^ AH bacteria/mL (AH/mL) were required to generate a significant immune response (Fig. 1B to H). This response was 4- to 8-fold higher with TLR4, TLR5, MyD88, IL-1β, IL-10, and mucin-5AC expression than in the non-AH control group. Moreover, a 32-fold increase in IL-8 was observed (Fig. 1F). Additionally, we found gradual but not threshold-dependent increases in IL-10 and mucin-5AC expression when comparing the 10^6^-AH/mL-challenged fish to the rest of the groups (Fig. 1G and H). To exclude the possibility that this immunostimulatory response was systemic, IL-1β and IL-10 expression levels were examined in the liver, spleen, and kidney of fish in the 10^6^-AH/mL group and compared to those in the non-AH control group. We found no significant difference in IL-1β and IL-10 expression levels between the two groups (see Fig. S1 in the supplemental material), indicating that immune stimulation was not systemic. Additionally, all fish in the two groups survived, without noticeable symptoms or the presence of AH in the liver, spleen, and kidney (Table S1). These data suggest that skin in the presence of mucus can be immune stimulated by continuous AH bath challenge.

10.1128/msphere.00194-22.2 FIG S1 Immune responses of the liver, kidney, and spleen to AH challenge. The expression levels of IL-1β and IL-10 in the liver (A), spleen (B), and kidney (C) were examined by qPCR in fish challenged with 10^6^ AH bacteria compared to nonchallenged control fish (*n* = 3 fish in each group). Statistical significance was determined by Student’s *t* test. Download FIG S1, TIF file, 2.1 MB. Copyright © 2022 Chen et al. 2022 Chen et al. https://creativecommons.org/licenses/by/4.0/ This content is distributed under the terms of the Creative Commons Attribution 4.0 International license.

10.1128/msphere.00194-22.7 TABLE S1 Examination of AH in the liver, spleen, and posterior kidney in both the control and 10^6^-AH/mL challenge groups. Download Table S1, DOCX file, 0.01 MB. Copyright © 2022 Chen et al. 2022 Chen et al. https://creativecommons.org/licenses/by/4.0/ This content is distributed under the terms of the Creative Commons Attribution 4.0 International license.

### AH-challenged skin mucus elicits similar immune stimulation in the *ex vivo* skin model.

**FIG 2:**
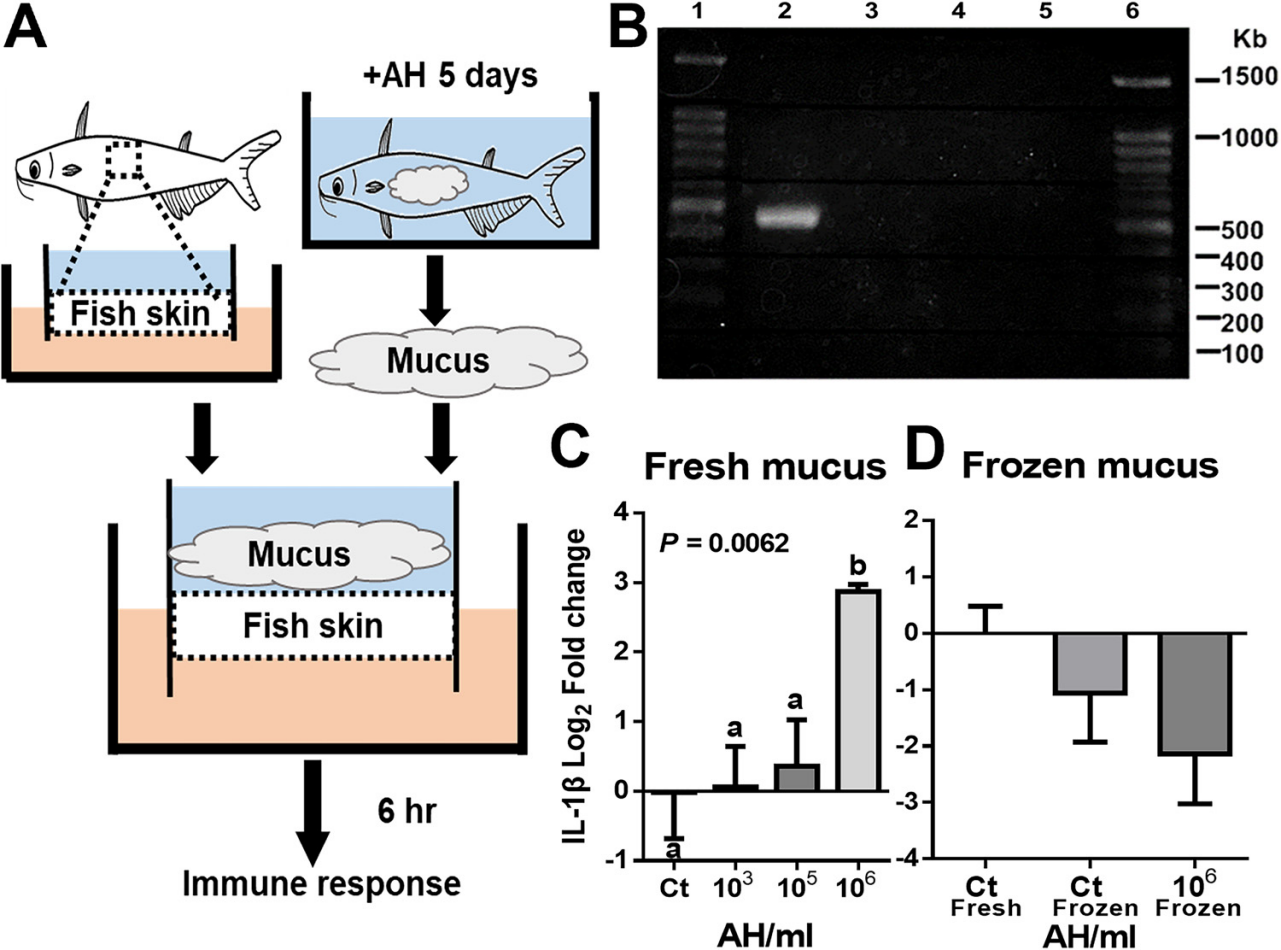
Fish skin immune response of the *ex vivo* skin model inoculated with mucus from AH-challenged fish. The skin mucus of AH-challenged and nonchallenged fish was scraped and transplanted to the *ex vivo* skin model for 6 h. qPCR of the skin tissue was performed to determine the IL-1β expression level. (A) Illustration of the experiment. (B) Confirmation of the gnotobiotic properties of the skin model by gel electrophoresis of the amplified 16S rRNA gene V3-V4 region (lanes 1 and 6, 10-kb ladder; lane 2, positive control [AH]; lane 3, negative control [double-distilled water]; lane 4, media from the apical and basal sides of the model; lane 5, skin from the model system). (C and D) IL-1β expression levels in the *ex vivo* skin with fresh mucus (C) and frozen mucus (D) from AH-challenged fish (*n* = 5 fish in each group). Statistical significance was determined by one-way ANOVA followed by Tukey’s *post hoc* test at a *P* value of <0.05 (if treatment groups share the same lowercase letter, then the differences between the groups are not statistically significant).

Since fish skin mucus plays a critical role in stimulating and regulating the immune response, we hypothesized that AH-challenged skin mucus could induce the observed skin immune response. To test this hypothesis, an *ex vivo* skin model (28) was used to create an interactive environment between the skin and the mucus (Fig. 2A). To first confirm that the skin model can be used as a gnotobiotic tissue model, PCR of the bacterial 16S rRNA gene V3-V4 region was performed on the culture medium and the skin explant in the skin model. We found no bacterial growth in either medium from the apical and basal sides of the model or the explant, compared to a positive 500-bp band in fresh skin mucus (Fig. 2B), suggesting that the skin model system can be used as a gnotobiotic tissue model. We then sought to examine the effects of mucus on the skin. Freshly collected skin mucus from AH-challenged and control fish was applied to the skin model system for 6 h, and subsequent qPCR was performed to determine the expression levels of IL-1β. We found an 8-fold increase in the expression level of IL-1β in the skin model transplanted with mucus from 10^6^-AH/mL group compared to the non-AH control, 10^3^-AH/mL, and 10^5^-AH/mL groups, whereas no significant change was found among the other comparisons (Fig. 2C). Additionally, the 10^2^-AH/mL group was excluded from this comparison due to unexpected contamination issues in the *ex vivo* skin model. To further examine whether the increased expression of IL-1β resulted from living biological properties, frozen mucus from the 10^6^-AH/mL group was examined. Without any growth of bacteria from frozen mucus (data not shown), we found no significant IL-1β expression changes in skin treated with frozen mucus compared to fresh mucus (Fig. 2D), indicating that living biological properties can cause immune stimulation. These data suggest that the living biological properties of AH-challenged skin mucus can directly stimulate the skin immune response, similar to the response observed *in vivo*. Therefore, mucus with its living biological properties under the interaction with AH may play a vital role in the health status of fish skin.

### Skin mucus mixed with AH does not stimulate the skin immune response.

**FIG 3:**
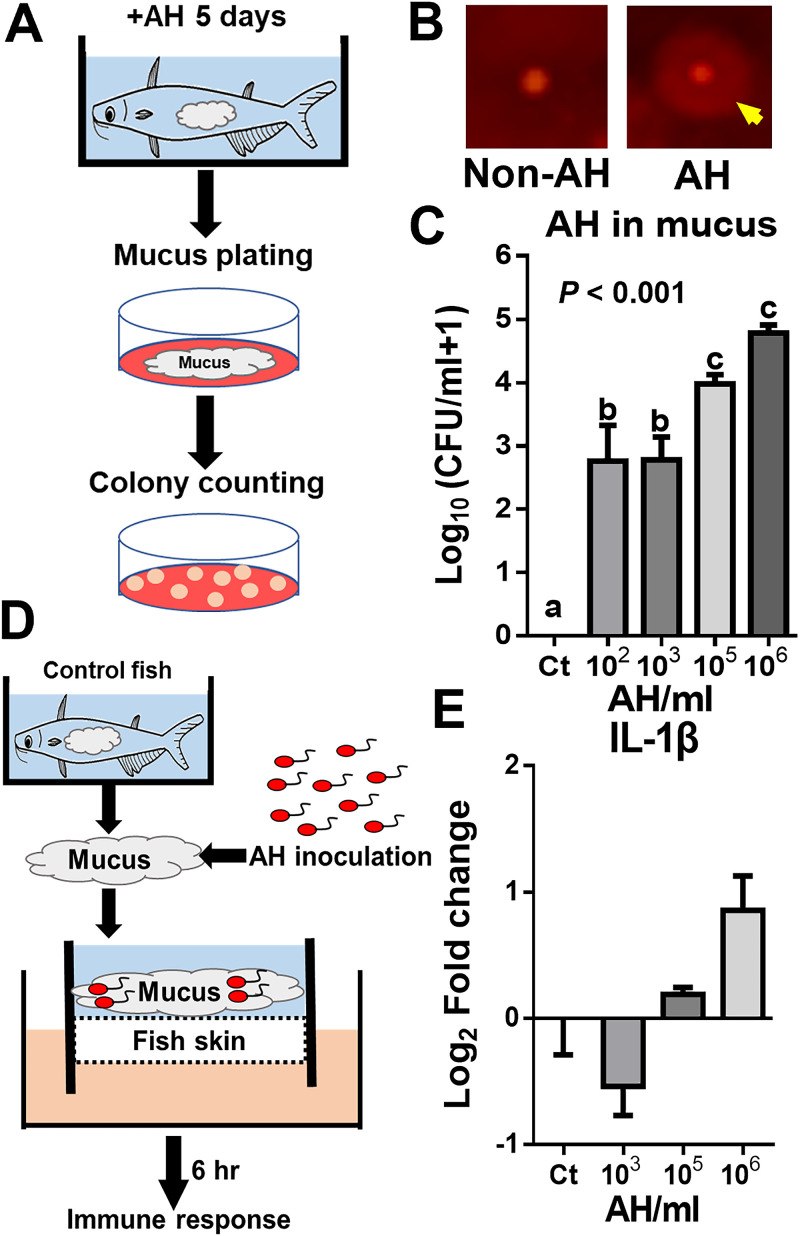
Viable AH in mucus and its contribution to the fish skin immune response in the *ex vivo* skin model. The viable AH concentration in mucus was quantified by directly plating collected skin mucus. (A) Illustration of viable AH quantification. (B) Starch hydrolysis of AH and non-AH bacteria in starch-ampicillin agar with an iodine solution. The yellow arrow indicates the clear halo surrounding the colony due to starch hydrolysis. (C) AH survival in mucus shown as CFU per milliliter of mucus (*n* = 8 fish in each group). Comparable numbers of AH bacteria were mixed into the mucus from nonchallenged fish and inoculated onto the skin of the *ex vivo* skin model for 6 h. qPCR was performed to determine the IL-1β expression level. Statistical significance was determined by one-way ANOVA followed by Tukey’s *post hoc* test at a *P* value of <0.05 (if treatment groups share the same lowercase letter, then the differences between the groups are not statistically significant). (D) Illustration of the AH-mucus mix in the *ex vivo* skin model. (E) IL-1β expression level in the *ex vivo* skin with the AH-mucus mix after a 6-h incubation (*n* = 5 fish in each group). Statistical significance was determined by one-way ANOVA followed by Tukey’s *post hoc* test at a *P* value of <0.05 (if treatment groups share the same lowercase letter, then the differences between the groups are not statistically significant).

The presence of AH has been shown to stimulate the skin immune response. However, whether AH in the mucus can stimulate the skin immune response is unknown. We addressed this question by inoculating different numbers of AH bacteria into mucus collected from nonchallenged fish skin and applying aliquots to the skin model. To mimic *in vivo* conditions, the viable AH concentration in the mucus was determined by diluting and plating freshly collected mucus from AH-challenged and control fish onto starch-ampicillin (SA) agar (Fig. 3A). The counting method was performed by adding an iodine solution to distinguish AH from other bacteria by starch hydrolysis around the AH colonies (Fig. 3B). We found viable AH bacteria in every challenged group but not the non-AH control group. The AH concentration in the mucus increased as the challenge concentration increased but was 5- to 10-fold lower than the concentration in the water tank. In addition, similar AH concentrations in the mucus were found at challenge concentrations of 10^2^ and 10^3^ AH/mL (Fig. 3C).

To examine whether the corresponding concentrations of AH in the mucus can induce a skin immune response, the desired number of AH bacteria was inoculated into skin mucus collected from nonchallenged fish to create an AH-mucus mix without changing the rest of the microbial community. The mucus was applied to the skin model and incubated for 6 h, followed by measuring the IL-1β expression level (Fig. 3D). We found no significant change in IL-1β levels in the skin model treated with an AH-mucus mix corresponding to the concentration *in vivo* compared to the skin model with control mucus (Fig. 3E). These data suggest that the concentration of AH in the mucus under our challenge concentration range alone may not contribute to the host skin response. Therefore, these data imply that the interactive biological properties, instead of AH alone, contribute to the skin immune response.

### Compositional changes in the skin mucosal microbiota by AH challenge.

**FIG 4:**
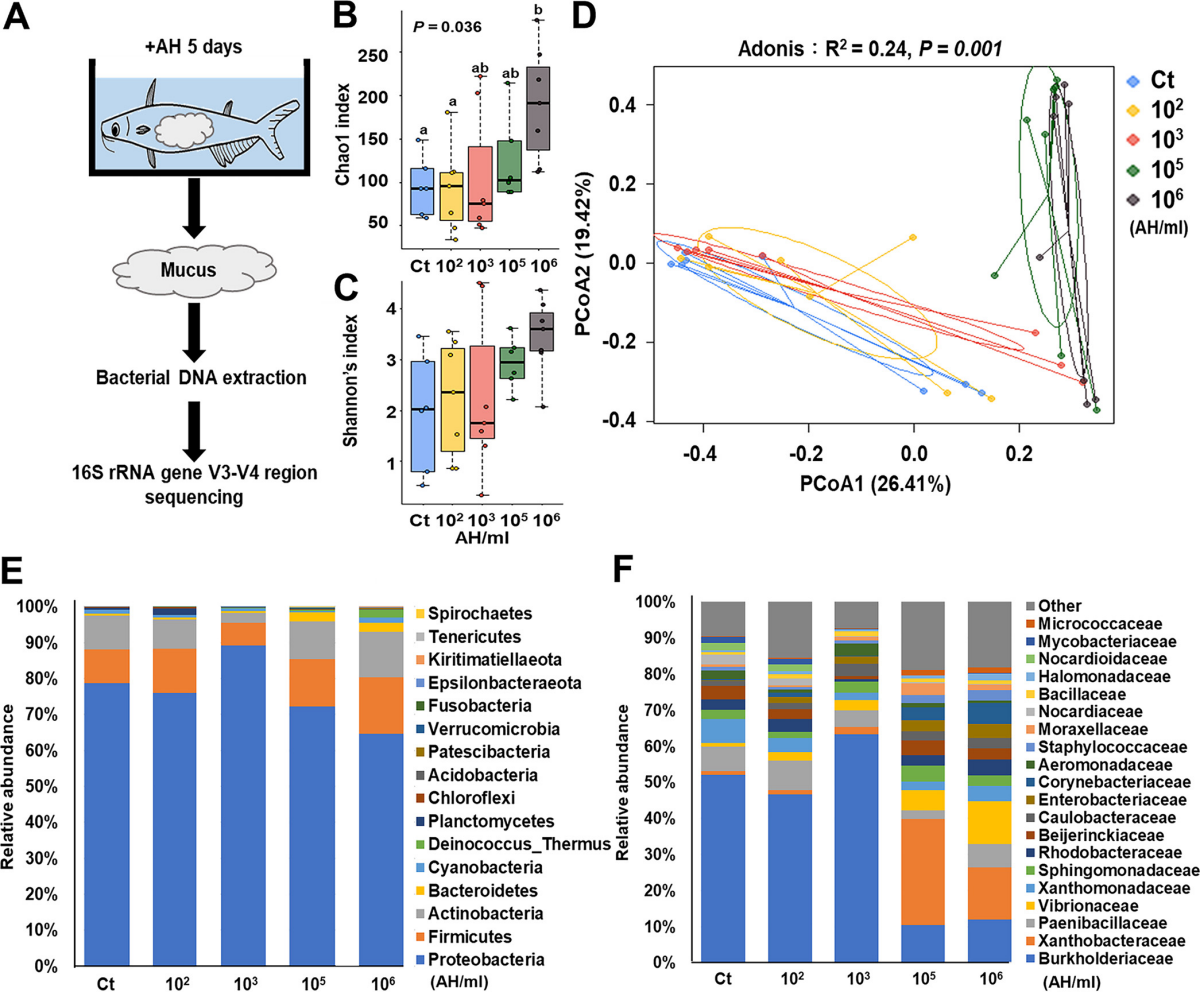
Changes in the skin mucosal microbiota after AH challenge. The skin mucus of AH-challenged and nonchallenged fish was scraped, and the microbial DNA was extracted, followed by 16S rRNA gene sequencing. (A) Illustration of the experiment. (B and C) Chao1 (B) and Shannon (C) alpha-diversity indices. Statistical significance was determined by one-way ANOVA followed by Tukey’s *post hoc* test at a *P* value of <0.05 (if treatment groups share the same lowercase letter, then the differences between the groups are not statistically significant). (D) Principal-coordinate analysis (PCoA) was performed at the ASV level based on Bray-Curtis dissimilarity and shows the distribution of the bacterial composition of individuals in each group (*P* = 0.001 by an Adonis test). (E and F) Relative abundances of members of the skin mucosal microbiota at the phylum level (E) and the family level (F) (*n* = 8 fish in each group).

Since the immunostimulatory effect was not elicited by mucus mixed with AH alone but was elicited by mucus from AH-challenged fish, we hypothesized that the skin mucosal microbiota underwent a significant change after AH challenge. To examine if the skin mucosal microbiota composition changed by AH challenge, the 16S rRNA gene V3-V4 regions of the collected skin mucus of individual fish from AH-challenged and non-AH control groups were sequenced to determine bacterial taxonomy, richness, and abundance (Fig. 4A). For alpha-diversity, we found that the 10^6^-AH/mL group, but not the other AH-challenged groups, had a significantly higher Chao1 index than the control group (Fig. 4B). However, we found a higher but insignificant Shannon index between the groups (Fig. 4C). For beta-diversity, principal-coordinate analysis (PCoA) was performed to examine the similarity among individuals and groups. In general, we found that individuals in the 10^2^- and 10^3^-AH/mL-challenged group clustered with the non-AH control group (Fig. 4D, left), whereas individuals in the 10^5^- and 10^6^-AH/mL-challenged groups clustered together but away from the non-AH control group (Fig. 4D, right). To further confirm the clustering difference in the PCoA, Adonis pairwise comparisons of microbiota compositions were applied based on Bray-Curtis distances (Table 1). These data suggest that the microbiota composition changed significantly in the 10^5^- and 10^6^-AH/mL-challenged groups. Thus, we denote the groups challenged with 10^5^ and 10^6^ AH/mL the high-concentration groups, whereas those challenged with 10^2^ and 10^3^ AH/mL are denoted the low-concentration groups. To examine the compositional differences in the microbiota between the two clustering groups and compared to the non-AH control group, the microbiota composition and abundance were classified at the phylum and family levels for comparison. At the phylum level, we found minimal differences, where the most abundant phyla in all samples were *Proteobacteria*, *Firmicutes*, and *Actinobacteria*, comprising more than 95% of the microbiota (Fig. 4E and Fig. S2A). However, a drastic difference was observed at the family level in the high-concentration groups compared to the other groups (Fig. 4F and Fig. S2B). The most abundant bacterial families in the non-AH control and low-concentration groups were similar, and *Burkholderiaceae*, *Paenibacillaceae*, and *Xanthomonadaceae* were present at abundances of 60 to 70%. However, in the high-concentration groups, we found 40 to 50% lower abundances of *Burkholderiaceae* but 12 to 28% higher abundances of *Xanthobacteraceae*, followed by a 5 to 10% increase in *Vibrionaceae* and 2 to 5% increases in *Enterobacteriaceae*, *Corynebacteriaceae*, *Moraxellaceae*, and *Caulobacteraceae*. Therefore, AH challenge induced significant changes in the skin mucosal microbiota at a high challenge concentration.

**TABLE 1:** Adonis pairwise comparisons of microbiota compositions between non-AH- and AH-challenged groups based on Bray-Curtis distances^*a*^

Challenge groups (AH/mL)	Adonis	
	*R* ^2^	*P* value
Ct vs 10^2^	0.043	0.74
Ct vs 10^3^	0.096	0.14
Ct vs 10^5^	0.26	**0.002**
Ct vs 10^6^	0.28	**0.002**
10^2^ vs 10^3^	0.051	0.62
10^2^ vs 10^5^	0.2	**0.002**
10^2^ vs 10^6^	0.21	**0.002**
10^3^ vs 10^5^	0.18	**0.009**
10^3^ vs 10^6^	0.19	**0.004**
10^5^ vs 10^6^	0.057	0.49

a Significant differences are highlighted in boldface type. Ct, control.

10.1128/msphere.00194-22.3 FIG S2 Skin mucosal microbiota composition of AH-challenged fish. The skin mucus of AH-challenged and nonchallenged fish was scraped, and the microbial DNA was extracted, followed by 16S rRNA gene sequencing. The relative abundances of members of the skin mucosal microbiota for individuals in each group are shown as percentages at the phylum level (A) and the family level (B) (*n* = 8 fish in each group). Download FIG S2, JPG file, 2.3 MB. Copyright © 2022 Chen et al. 2022 Chen et al. https://creativecommons.org/licenses/by/4.0/ This content is distributed under the terms of the Creative Commons Attribution 4.0 International license.

### Microbial composition changes at high challenge concentrations alter the functional capacity and metabolic output.

**FIG 5:**
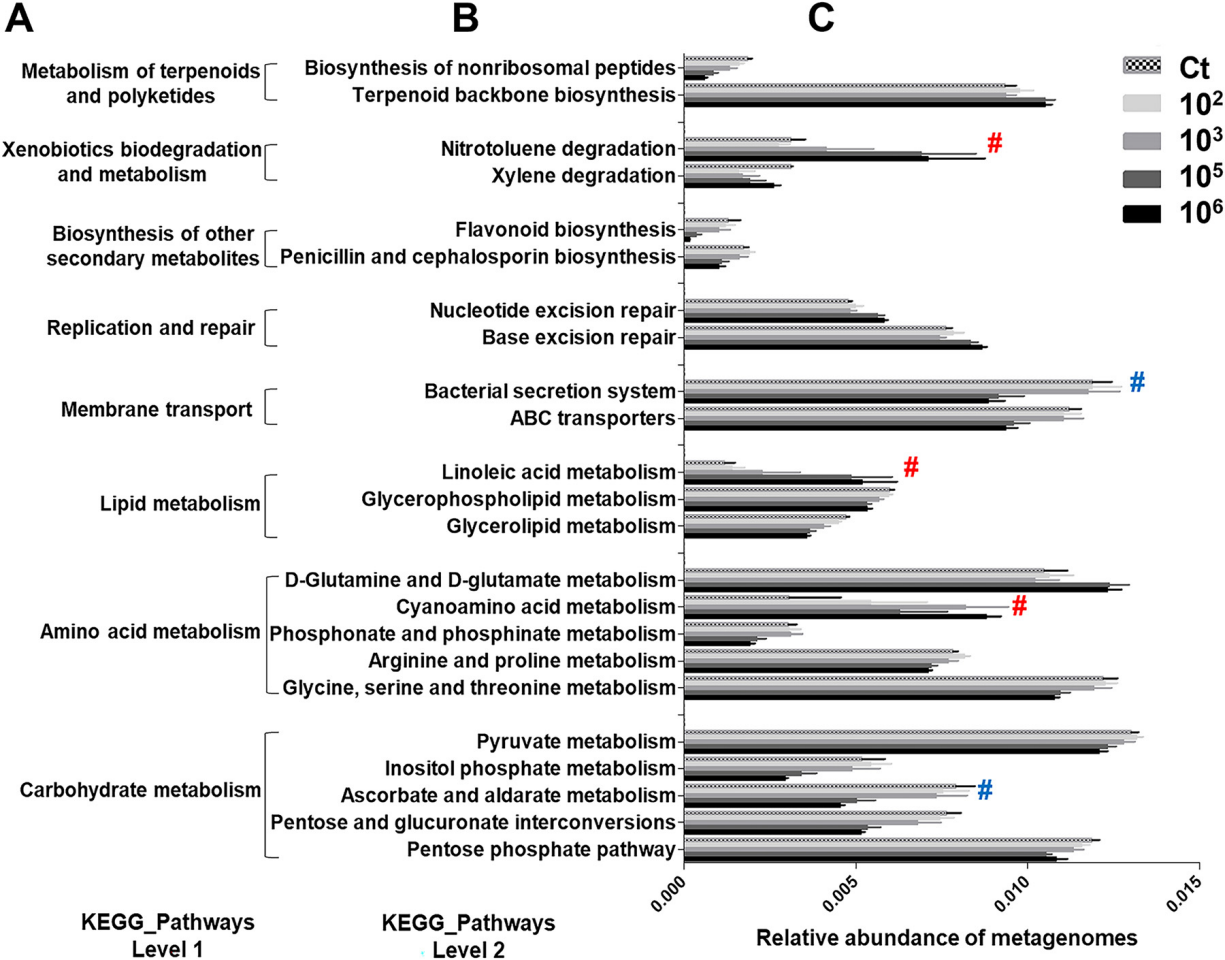
Changes in the metabolic and functional outputs of the skin mucosal microbiota after AH challenge. Functional prediction of the microbiota was performed by PICRUSt2 and annotated using the KEGG database. (A and B) Significant differences in level 1 (A) and level 2 (B) KEGG pathways. (C) Abundance of each functional pathway (*n* = 8 fish in each group). Statistical significance was determined by one-way ANOVA followed by Tukey’s *post hoc* test (#, both groups challenged with 10^5^ and 10^6^ AH/mL were both significantly different at a *P* value of <0.05 compared to the other groups; red # and blue # indicate up- and downregulated pathways, respectively).

When the microbial composition changes, the metabolic output and functional capacity of the community may also be altered (29). To determine if the metabolic output and/or functional capacity changed in the skin mucus microbiota, Phylogenetic Investigation of Communities by Reconstruction of Unobserved States (PICRUSt2) analysis using the Kyoto Encyclopedia of Genes and Genomes (KEGG) database was performed to compare the microbiota metabolic outputs and functions in different AH challenge groups to those in the non-AH control group. The significant differences and abundances of KEGG pathways analyzed by one-way analysis of variance (ANOVA) are summarized in Fig. 5. The level 1 pathways (Fig. 5A) indicated general metabolic outputs such as the metabolism of terpenoids, polyketides, xenobiotics, lipid, amino acid, and carbohydrates; the biosynthesis of other secondary metabolites; nucleotide replication; repair; and membrane transport. Significant differences in the relative abundances of subcategorized level 2 pathways (Fig. 5B and C) were determined by one-way ANOVA followed by Tukey’s *post hoc* test, and we required the 10^5^- and 10^6^-AH/mL-challenged groups to have significant differences at a *P* value of <0.05 compared to the other groups. We found significant increases in the relative abundances of pathways related to linoleic acid and cyanoamino acid metabolism and nitrotoluene degradation but significant decreases in the relative abundances of pathways related to the bacterial secretion system and ascorbate and aldarate metabolism, suggesting that the functional capacity and metabolic output of the skin mucus microbiota were altered by AH challenge.

### The increase in opportunistic pathogens in the mucosal microbiota potentially stimulates the skin immune response.

**FIG 6:**
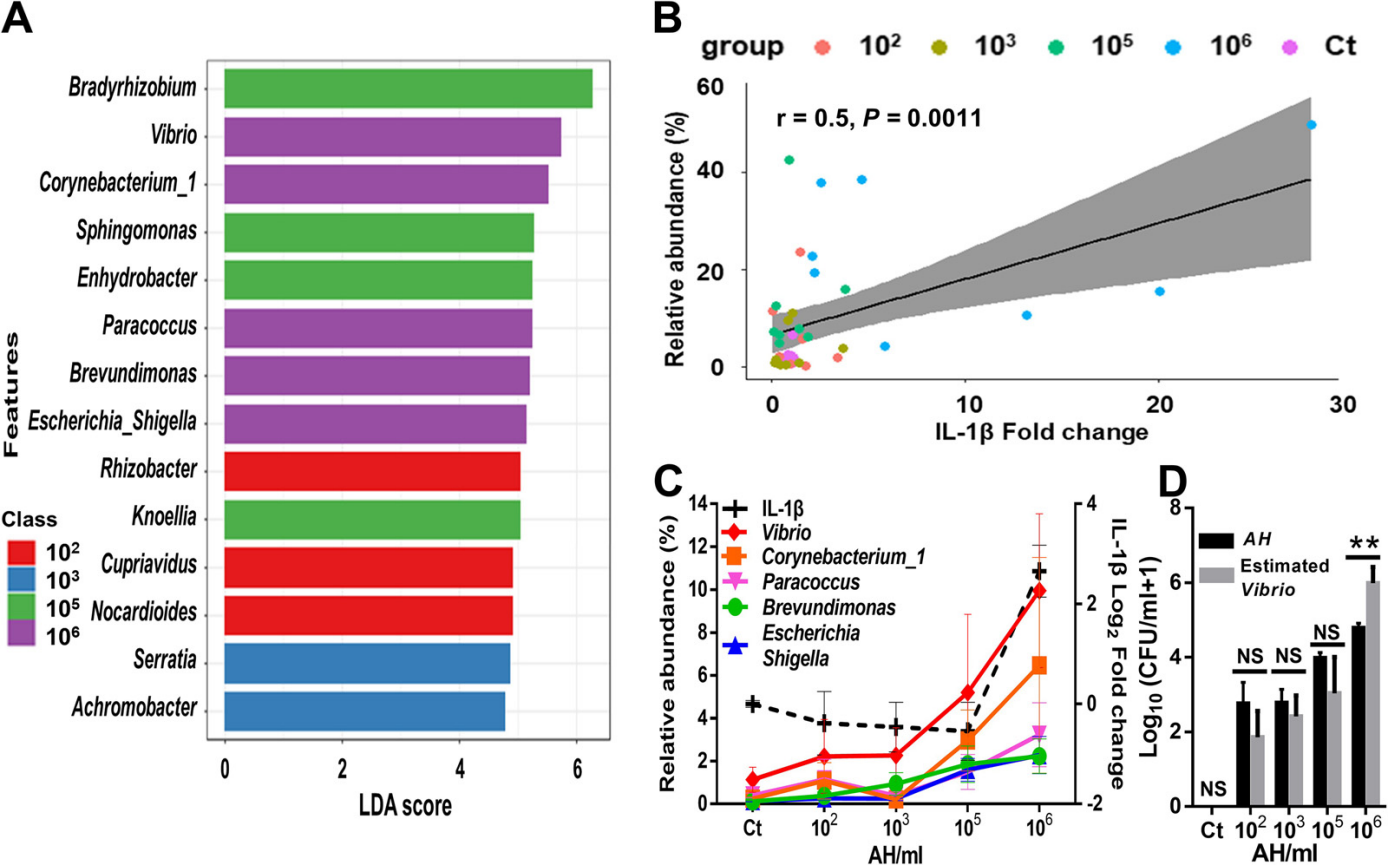
An increase in opportunistic pathogens is correlated with skin immune stimulation. The skin mucosal microbiota composition of the 10^6^-AH/mL group was compared to those of the rest of the groups. (A) LEfSe analysis showing differentially abundant genera as biomarkers determined using a Kruskal-Wallis test (*P* value cutoff of 0.1) with an LDA score of 2. (B) Correlation between IL-1β expression levels and increased skin mucosal microbiota genera in 10^6^-AH/mL-challenged fish. (C) Relative abundance trend of increased genera in the 10^6^-AH/mL group followed by increased AH challenge concentrations. (D) Comparison of the concentration of viable Aeromonas hydrophila and the estimated concentration of *Vibrio* in mucus under different AH challenge concentrations (*n* = 8 fish in each group). Statistical significance was determined by Student’s *t* test (***, *P* ≤ 0.001; **, *P* ≤ 0.01; *, *P* ≤ 0.05; NS, not significant).

Composition and functional analyses have shown significant differences in the skin mucosal microbiota between low- and high-AH-concentration challenge groups. However, immune marker expression levels significantly increased only at 10^6^ AH/mL in the high-AH-concentration challenge groups *in vivo* and *ex vivo*. We hypothesized that there was a difference in the skin mucosal microbiota between 10^5^- and 10^6^-AH/mL-challenged groups in determining the stimulation of the skin immune response. Since the two groups were not significantly different in the Bray-Curtis matrix, we specified the microbiota differences by performing linear discriminant analysis (LDA) effect size (LEfSe) analysis to show the unique niches of the bacterial groups in the 10^6^-AH/mL-challenged skin mucosal microbiota compared to the rest of the groups. We found a unique niche of *Vibrio*, *Corynebacterium*, *Paracoccus*, *Brevundimonas*, and Escherichia*-Shigella* at 10^6^ AH/mL (Fig. 6A). We correlated these unique bacterial genera to the increase in the IL-1β expression level and found a significant positive correlation (*r* = 0.5; *P* = 0.0011) (Fig. 6B). Moreover, we found that the relative abundances of these genera were gradually increased when the challenge concentration increased to 10^6^ AH/mL (Fig. 6C). Among these genera, *Vibrio* had the highest and most significant increase, from 1% in the non-AH control group to 5% in the 10^5^-AH/mL and 10% in the 10^6^-AH/mL groups (Table S2). Other genera, such as *Corynebacterium*_1, *Paracoccus*, *Brevundimonas*, and Escherichia*-Shigella*, had gradual increases from 2 to 4% relative abundances from the low- to the high-AH-challenge groups. We hypothesized a 10^6^-AH/mL challenge concentration could induce the growth of the genus *Vibrio* over a threshold concentration to stimulate IL-1β expression. To estimate the concentration of *Vibrio* in mucus, viable AH bacteria from plating were used and applied to the ratio of AH to *Vibrio* from relative abundances based on 16S rRNA gene sequencing data for individual fish. We then compared the concentration of viable AH and the estimated concentration of *Vibrio* in the skin mucus of each fish in different groups and found that the estimated *Vibrio* concentration was significantly 10-fold higher than that of AH in the 10^6^-AH/mL group, whereas a nonsignificant difference was observed in the other groups (Fig. 6D). The data imply that *Vibrio* bacteria can potentially increase to numbers higher than those of AH in the mucus and potentially be the stimulant of the skin immune response. Taken together, the increase in the AH concentration can concomitantly increase opportunistic pathogens, which may play a primary role over AH in stimulating the host immune response.

10.1128/msphere.00194-22.8 TABLE S2 Statistical analysis to determine significant differences in the relative abundances of bacterial genera between each AH-challenged group and the non-AH control group (***, *P ≤ *0.001; **, *P ≤ *0.01; *, *P ≤ *0.05; NS, no significance [by one-way ANOVA followed by Dunnett’s *post hoc* test]). Download Table S2, DOCX file, 0.01 MB. Copyright © 2022 Chen et al. 2022 Chen et al. https://creativecommons.org/licenses/by/4.0/ This content is distributed under the terms of the Creative Commons Attribution 4.0 International license.

## DISCUSSION

**FIG 7:**
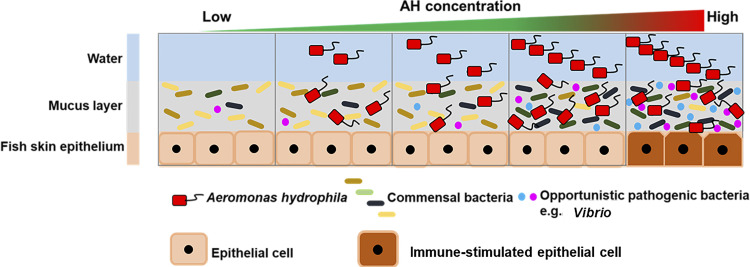
Prospective model of interactions among the host skin, mucosal microbiota, and AH. The results suggest that the fish skin mucosal microbiota composition is maintained and presents homeostasis in the mucosal layer in the presence of a low concentration of AH. With an increased concentration of AH, the skin mucosal microbiota composition changes but still maintains homeostasis in the mucosal layer. Once the AH concentration increases over a critical threshold where opportunistic pathogens increase in the changed microbiota, dysbiosis occurs in the mucosal layer, thus stimulating an immune response in the skin.

Initial interactions among pathogens, the skin mucosal microbiota, and host skin are essential but largely unexplored. In this study, we showed that the presence and increase of AH result in a dysbiotic mucosal microbiota that can stimulate the immune response of the skin (Fig. 7).

Previous studies have shown that fish skin mucus is essential for regulating skin immunity and protecting fish from pathogen invasion (30). In our study, performing *in vivo* experiments and using an *ex vivo* skin model, we found a stimulated immune response in the skin model inoculated with fresh challenged mucus (Fig. 2C) but not unchallenged mucus mixed with AH or frozen mucus (Fig. 2D and Fig. 3E). Our previous study showed a significant immunostimulatory effect when inoculating the same amount of AH directly onto the skin model without mucus (28). Therefore, striped catfish skin mucus has a protective role in response to AH. A similar observation has been reported for blue and channel catfish where infection occurred when the skin mucus was abraded/scraped off under bath challenge (26, 27). Several studies suggested that the skin mucus possesses host-secreted antimicrobial properties that protect the host from pathogens (31, 32). We have explored the anti-AH properties of the skin mucus in striped catfish but found no effect against AH growth (see Table S3 in the supplemental material). A similar observation has been made using apical medium containing mucus secretions from the *ex vivo* model (data not shown). This suggests that skin mucus possesses an immune-buffering rather than a growth-inhibitory role against AH, potentially derived from the microbiota. Two possible mechanisms can be hypothesized. First, the skin mucosal microbiota can directly buffer the immune response by interacting with AH. Several bacteria can serve as potential candidates in this buffering system by colony resistance and/or the production of adherence-inhibitory compounds (33–35). Second, previous studies have shown that the fish commensal microbiota can regulate the host immune system (12, 36–39). An immunostimulatory role was also found in striped catfish skin mucus using the *ex vivo* skin model (Fig. S3). Multiple bacterial genera can stimulate and boost the host immune system for responding to pathogens faster. These genera include *Lactobacillus*, *Shewanella*, and *Bacillus* (40–42). Even though these genera are not in the amplicon sequence variant (ASV) table of our research, changing the properties of the mucosal microbiota may be enough to induce immune stimulation (13). It would be interesting to manipulate the mucosal microbiota composition using mucus isolates and metabolism under different stress conditions to examine how the mucosal microbiota can change the skin immune response.

10.1128/msphere.00194-22.4 FIG S3 Immune response of striped catfish skin to mucus. Fresh mucus was scraped and transplanted to the *ex vivo* skin model. After a 6-h challenge, fish skin was collected, and qPCR was performed to determine the IL-1β expression level. Statistical significance was determined by Student’s *t* test. (*, *P ≤ *0.05). Download FIG S3, TIF file, 1.0 MB. Copyright © 2022 Chen et al. 2022 Chen et al. https://creativecommons.org/licenses/by/4.0/ This content is distributed under the terms of the Creative Commons Attribution 4.0 International license.

10.1128/msphere.00194-22.9 TABLE S3 Anti-AH properties of skin mucus were examined with different treatments, including the positive control of tetracycline, the negative control of double-distilled water, and different concentrations of mucus. Download Table S3, DOCX file, 0.01 MB. Copyright © 2022 Chen et al. 2022 Chen et al. https://creativecommons.org/licenses/by/4.0/ This content is distributed under the terms of the Creative Commons Attribution 4.0 International license.

From the *in vivo* and *ex vivo* models, we have gained mechanistic information on how AH challenge induces an increase in pathogens in the mucosal microbiota that can stimulate the immune response of the skin (Fig. 7). Here, we found that *Vibrio*, which has been reported to be an important opportunistic pathogen responsible for aquatic infectious diseases (43–45), is drastically increased in the skin mucus of 10^6^-AH/mL-challenged fish. This suggests that AH can directly or indirectly trigger the growth of opportunistic pathogens. Rolig et al. showed that the presence of AH can increase the number of *Vibrio* bacteria and attract neutrophils to the gut of gnotobiotic zebrafish (38). These data support the hypothesis that AH can directly help increase the number of *Vibrio* bacteria. Zhang et al. also showed that the presence of parasitic pathogen infection can influence the skin microbiota and change the skin mucus into an opportunistic-pathogen-favoring environment in rainbow trout (46). However, another hypothesis is that the host contributes to the increase in *Vibrio*. Studies have shown that teleost stress itself can influence the skin microbiome and change the skin mucus into an opportunistic-pathogen-favoring environment in brook trout (47). Both mechanisms can occur individually or synergistically on fish skin. In addition to *Vibrio*, we observed that other genera were increased at 10^6^ AH/mL compared to 10^5^ AH/mL. *Corynebacterium*_1, *Paracoccus*, *Brevundimonas*, and Escherichia*-Shigella* were present in the low-concentration challenge group or the non-AH control group and then gradually increased when the challenge concentration increased. Previous studies have shown that these bacteria can be opportunistic aquatic pathogens. *Corynebacterium*_1 was isolated from diseased striped bass (48), Escherichia*-Shigella* bacteria are common pathogens in the gastrointestinal tract of diseased fish (49), and *Brevundimonas* has recently been defined as an emerging global opportunistic pathogen (50). Therefore, it is reasonable to postulate that mucus changed by AH challenge might become an environment for these opportunistic pathogenic bacterial genera to become noticeable in abundance. In addition, PICRUSt2 analysis of metabolic profiles also showed that the mucus environment in the high-concentration-challenged groups favors a microbiota with nucleotide repair and linoleic acid metabolism (Fig. 5), implying an unfavorable environment for microbial growth (51, 52). With *Vibrio* and other halophiles growing, a more highly osmotic environment can be hypothesized to be present in the skin mucus of the high-concentration-challenged fish. Previous studies have found that the presence of pathogens or other stresses can regulate fish skin osmolality, driving salt secretion (53). It would be interesting to examine Na^+^/K^+^-ATPase activity and salt density in mucus to correlate them with the extremophiles that we found. Taken together, the tripartite interaction of the pathogen, the skin, and the skin mucosal microbiota is essential and inseparable when interpreting infection and inflammation.

Despite the broad information that this work provides on this tripartite interaction prior to or at the initial stage of AH infection, there are still several limitations of this work. First, aquaculture environmental and seasonal factors can constantly change the skin mucosal microbiota (54), meaning that the microbiota in the experimental fish in this study may represent only a niche in many cases. Previous studies have shown that the fish skin mucosal microbiota is represented by major phyla, including *Proteobacteria*, *Firmicutes*, *Actinobacteria*, *Bacteriodetes*, and *Cyanobacteria* (55, 56). Our work has shown the same trend in phyla (Fig. 4F). However, this trend can be different at the family and genus levels. Thus, our work may apply to only one of several scenarios. Second, the resolution of 16S rRNA V3-V4 gene sequencing is limited to the genus but not the species level. The actual species changes in targeted opportunistic pathogens will rely on their isolation (i.e., *Vibrio*) along with further sequencing and *ex vivo* skin model examination. Moreover, the individual differences in *in vivo* experiments could hinder the interpretation of data from 16S-based analyses (i.e., PCoA and LEfSe). Increasing the sample size and examining outliers should be considered for future experiments. Third, based on our data on increased IL-8 expression, neutrophils may be recruited. A previous study has shown that immune cells work coordinately with epithelial cell signals to clear pathogens (57). In the future, how the immune cells in the skin work can be explored by adding immune cells to our skin model. Fourth, immune expression is the sum of the balance between immune stimulation and inhibition. Even though immune stimulation was found only in the 10^6^-AH/mL group after the 5-day experiment, the regulation of the immune system during the experiment and in the other groups was still unknown. Fluctuating expression of innate immune markers in blue catfish was found in the first 12 to 24 h after AH challenge, showing a fast immune response to the presence of AH (27). It would be interesting to examine immune regulation in a time-dependent manner during challenge in the future.

In the future, we will explore the mutual interactions of each participant in this complicated interaction. On the bacterium side, the immunostimulatory roles of mucosal commensals and mechanistic information on how AH induces an increase in opportunistic pathogens are of interest. On the host side, how the immune system is coordinated with epithelial cells and mucus-secreting cells in response to the mucosal microbiota changes induced by pathogens will need to be further understood. We believe that this work has implications for aquaculture management regarding the presence of environmental pathogens and can shed some light on early aquaculture infection.

## MATERIALS AND METHODS

### Experimental fish and model overview.

Striped catfish (*Pangasianodon hypophthalmus*) were obtained from a local aquarium vendor and were bred and maintained in a 300-L tank at 28°C in the aquaculture room at the Department of Marine Biotechnology and Resources. A 12-h/12-h light/dark period was maintained, and an air stone supplied supplemental aeration. After breeding, eight fish with an average weight of 23 g were transferred to separate 80-L tanks (40 L of water) by experimental treatments and acclimated for half a month. Tap water was used as our rearing water source, and microbial growth tests were regularly performed to ensure that there was no growth of bacteria. After acclimation, fish in each tank were challenged with different concentrations of the pathogen Aeromonas hydrophila (AH) for 5 days. An overview of the experimental treatments and transitions is given in Fig. S4 in the supplemental material The water temperature during challenge was controlled at 28°C. The experiment was permitted by the Institutional Animal Care and Use Committee (IACUC) of National Sun Yet-Sen University with approval numbers 10834 and 10836.

10.1128/msphere.00194-22.5 FIG S4 Experimental design and frameworks. Striped catfish were challenged with different concentrations of AH. After challenge, the skin was removed and examined for immune responses by qPCR. The mucus was used for microbiota examination by 16S rRNA gene sequencing analysis, viable AH counts were determined by AH plating, and the skin immune response was reconfirmed by mucus transplantation into the *ex vivo* skin model system. Download FIG S4, JPG file, 0.6 MB. Copyright © 2022 Chen et al. 2022 Chen et al. https://creativecommons.org/licenses/by/4.0/ This content is distributed under the terms of the Creative Commons Attribution 4.0 International license.

### Aeromonas hydrophila challenge.

AH bacteria were cultured from a single isolate (AH20) (catalog number 16704; BCRC, Hsinchu, Taiwan), inoculated onto multiple starch-ampicillin agar plates (catalog number M1177; HiMedia, India), and incubated in an incubator at 28°C overnight. AH bacteria were then swabbed, suspended in tap water, and adjusted to the desired concentration for inoculation into experimental tanks. The concentration of AH in each tank was determined as CFU per milliliter by plating 100 μL of 10-fold serial dilutions onto SA agar plates every day 1 and 24 h after inoculation. To maintain the desired concentration in the 5-day challenge period, tank water was changed and reinoculated with AH every day (Fig. S5A to E). On sampling day 5, the tank water microbial composition was analyzed to confirm the relative abundance of AH in each experimental treatment (Fig. S5F and G).

10.1128/msphere.00194-22.6 FIG S5 AH concentration in the 5-day bath challenge period and microbial composition of tank water on day 5. The AH concentration in water was measured by plating tank water onto SA agar during the 5-day experiment period at two time points, 1 and 24 h after inoculation. On day 5, the microbial DNA of tank water was extracted, followed by 16S rRNA gene sequencing. Shown are AH concentrations in the control (A), 10^2^-AH/mL (B), 10^3^-AH/mL (C), 10^5^-AH/mL (D), and 10^6^-AH/mL (E) groups, and the relative abundances of the members of the microbiota in experimental tank water at day 5 in each group are shown as percentages at the phylum level (F) and the family level (G). Statistical significance was determined by one-way ANOVA followed by Tukey’s *post hoc* test. Download FIG S5, JPG file, 1.1 MB. Copyright © 2022 Chen et al. 2022 Chen et al. https://creativecommons.org/licenses/by/4.0/ This content is distributed under the terms of the Creative Commons Attribution 4.0 International license.

### Skin tissue and mucus collection.

Fish were collected from the control and treatment tanks after the 5-day AH challenge. After that, anesthesia was performed using rapid chilling followed by cervical transection. The sampling procedures were conducted according to *AVMA Guidelines for the Euthanasia of Animals* (https://www.avma.org/resources-tools/avma-policies/avma-guidelines-euthanasia-animals) (58). Skin mucus was removed by gentle scraping by slide. Fish skin tissue, followed by the liver, spleen, and kidney, was then removed, flash-frozen in liquid nitrogen during collection, and stored at −80°C until RNA extraction.

### Mucus transplantation.

A striped catfish model system based on our previous study (28) was applied in this research. Fish skin tissue was first removed from the fish for making the *ex vivo* skin model and cultured in a CO_2_-free incubator at 25°C for 5 days. The detailed protocol is described in Text S1. Twenty microliters of skin mucus scraped from fish in the challenge experiment, or regular skin mucus mixed with the desired concentration of AH in 2 μL was transplanted to the upper side of the fish skin model system, with double-distilled water on the apical side and medium on the basal side. After 6 h of mucus transplantation, the fish skin tissue was collected, followed by RNA extraction. A cDNA library was built and applied for qPCR to quantify the desired immune markers.

10.1128/msphere.00194-22.1 TEXT S1 Supplemental materials and methods. Download Text S1, DOCX file, 0.02 MB. Copyright © 2022 Chen et al. 2022 Chen et al. https://creativecommons.org/licenses/by/4.0/ This content is distributed under the terms of the Creative Commons Attribution 4.0 International license.

### RNA extraction and real-time PCR analyses.

Total RNA samples from the skin, liver, spleen, and kidney of striped catfish were extracted with TriPure isolation reagent (Roche, Mannheim, Germany) according to instructions described previously (59). A cDNA library was built using Moloney murine leukemia virus (M-MLV) reverse transcriptase (Promega, USA) according to the manufacturer’s instructions. All the cDNA products were utilized for quantitative real-time PCR using GoTaq qPCR master mix on a CFX96 real-time PCR detection system (Bio-Rad, CA). Primer pairs were designed using the Universal Probe library website and are shown in Table S4. The comparative threshold cycle (*C_T_*) (ΔΔ*C_T_*) method was used to evaluate the expression of candidate genes (60). The detailed protocol is shown in Text S1.

10.1128/msphere.00194-22.10 TABLE S4 Primer pairs used in this research. Download Table S4, DOCX file, 0.01 MB. Copyright © 2022 Chen et al. 2022 Chen et al. https://creativecommons.org/licenses/by/4.0/ This content is distributed under the terms of the Creative Commons Attribution 4.0 International license.

### Skin mucus microbial DNA extraction, 16S rRNA gene sequencing, and community analyses.

Skin mucus microbial DNA was extracted. After preparation and quality control, the 16S rRNA V3-V4 hypervariable region was sequenced on the Illumina MiSeq platform. The Quantitative Insights into Microbial Ecology (QIIME2.2019.10) software system was used to conduct quality filtering of raw data. The DADA2 method for ASV inference was used to process the 16S rRNA gene amplicon data. The Silva database classifier was applied for taxonomic assignment (61). Microbial community analyses were conducted with the RStudio (version 4.1.1) vegan package (62) as well as MicrobiomeAnalyst (63, 64). Functional prediction was conducted by PICRUSt2 using previously described scripts (65). The detailed protocol is described in Text S1.

### Quantification of viable AH in mucus.

The skin mucus was scraped after AH challenge. Twenty microliters of mucus was mixed with 180 μL followed by serial 10-fold dilutions and plated onto SA agar plates. After being incubated at 28°C overnight, the colonies were counted to calculate the actual AH concentration in mucus. An iodine solution (300 μg/mL) was added to the plate to distinguish AH from other bacteria by starch hydrolysis.

### Statistical analysis.

Statistical significance was assessed using Student’s *t* test and one-way analysis of variance (ANOVA) followed by Tukey’s honestly significant difference (HSD) multiple-comparison or Dunnett’s *post hoc* test. All data were confirmed to fit into a Gaussian distribution by a Shapiro-Wilk test for normality, and analyses were performed using Prism8 software (GraphPad Software, La Jolla, CA).

### Data availability.

Sequencing data have been submitted to the NCBI Sequence Read Archive (SRA) under accession number SRP352833.

## References

[1] Ángeles Esteban M. 2012. An overview of the immunological defenses in fish skin. ISRN Immunol 2012:853470. doi:10.5402/2012/853470.

[2] Peatman E, Lange M, Zhao H, Beck BH. 2015. Physiology and immunology of mucosal barriers in catfish (*Ictalurus* spp.). Tissue Barriers 3:e1068907. doi:10.1080/21688370.2015.1068907. 26716071 PMC4681283

[3] Ángeles Esteban M, Cerezuela R. 2015. Fish mucosal immunity: skin, p 67–92. *In* Beck BH, Peatman E (ed), Mucosal health in aquaculture. Academic Press, San Diego, CA.

[4] Tiralongo F, Messina G, Lombardo BM, Longhitano L, Li Volti G, Tibullo D. 2020. Skin mucus of marine fish as a source for the development of antimicrobial agents. Front Mar Sci 7:54.

[5] Gomez D, Sunyer JO, Salinas I. 2013. The mucosal immune system of fish: the evolution of tolerating commensals while fighting pathogens. Fish Shellfish Immunol 35:1729–1739. doi:10.1016/j.fsi.2013.09.032. 24099804 PMC3963484

[6] Dash S, Das SK, Samal J, Thatoi HN. 2018. Epidermal mucus, a major determinant in fish health: a review. Iran J Vet Res 19:72–81. 30046316 PMC6056142

[7] Tasleem F, Amjad R, Sherazi S, Saddique A, Saba A, Zainab S, Masood S. 2020. Physiology and biochemical properties of fish mucus particular emphasizes as a body defense system. Saudi J Biol Sci 5:274–280. doi:10.36348/sjls.2020.v05i12.002.

[8] Belkaid Y, Harrison OJ. 2017. Homeostatic immunity and the microbiota. Immunity 46:562–576. doi:10.1016/j.immuni.2017.04.008. 28423337 PMC5604871

[9] Arrieta MC, Finlay BB. 2012. The commensal microbiota drives immune homeostasis. Front Immunol 3:33. doi:10.3389/fimmu.2012.00033. 22566917 PMC3341987

[10] McGuckin MA, Lindén SK, Sutton P, Florin TH. 2011. Mucin dynamics and enteric pathogens. Nat Rev Microbiol 9:265–278. doi:10.1038/nrmicro2538. 21407243

[11] Kelly C, Salinas I. 2017. Under pressure: interactions between commensal microbiota and the teleost immune system. Front Immunol 8:559. doi:10.3389/fimmu.2017.00559. 28555138 PMC5430139

[12] Pérez-Pascual D, Vendrell-Fernández S, Audrain B, Bernal-Bayard J, Patiño-Navarrete R, Petit V, Rigaudeau D, Ghigo J-M. 2021. Gnotobiotic rainbow trout (*Oncorhynchus mykiss*) model reveals endogenous bacteria that protect against *Flavobacterium columnare* infection. PLoS Pathog 17:e1009302. doi:10.1371/journal.ppat.1009302. 33513205 PMC7875404

[13] Hapfelmeier S, Lawson MA, Slack E, Kirundi JK, Stoel M, Heikenwalder M, Cahenzli J, Velykoredko Y, Balmer ML, Endt K, Geuking MB, Curtiss R, III, McCoy KD, Macpherson AJ. 2010. Reversible microbial colonization of germ-free mice reveals the dynamics of IgA immune responses. Science 328:1705–1709. doi:10.1126/science.1188454. 20576892 PMC3923373

[14] Gomez de Agüero M, Ganal-Vonarburg SC, Fuhrer T, Rupp S, Uchimura Y, Li H, Steinert A, Heikenwalder M, Hapfelmeier S, Sauer U, McCoy KD, Macpherson AJ. 2016. The maternal microbiota drives early postnatal innate immune development. Science 351:1296–1302. doi:10.1126/science.aad2571. 26989247

[15] Bergmann KR, Liu SXL, Tian R, Kushnir A, Turner JR, Li H-L, Chou PM, Weber CR, De Plaen IG. 2013. Bifidobacteria stabilize claudins at tight junctions and prevent intestinal barrier dysfunction in mouse necrotizing enterocolitis. Am J Pathol 182:1595–1606. doi:10.1016/j.ajpath.2013.01.013. 23470164 PMC3644725

[16] Hooper LV, Littman DR, Macpherson AJ. 2012. Interactions between the microbiota and the immune system. Science 336:1268–1273. doi:10.1126/science.1223490. 22674334 PMC4420145

[17] Hu C, Huang Z, Liu M, Sun B, Tang L, Chen L. 2021. Shift in skin microbiota and immune functions of zebrafish after combined exposure to perfluorobutanesulfonate and probiotic *Lactobacillus rhamnosus*. Ecotoxicol Environ Saf 218:112310. doi:10.1016/j.ecoenv.2021.112310. 33971395

[18] Naylor RL, Hardy RW, Buschmann AH, Bush SR, Cao L, Klinger DH, Little DC, Lubchenco J, Shumway SE, Troell M. 2021. A 20-year retrospective review of global aquaculture. Nature 591:551–563. doi:10.1038/s41586-021-03308-6. 33762770

[19] Pridgeon J. 2012. Major bacterial diseases in aquaculture and their vaccine development. CAB Rev 7:11–16.

[20] Subhan U, Mauladani S, Rosidah, Bangkit I, Joni IM. 2020. Promising application of fine bubbles (FBs) to control *Aeromonas hydrophila* on striped catfish seed (*Pangasianodon hypophthalmus*). AIP Conf Proc 2020:e090002.

[21] Crumlish M, Thanh PC, Koesling J, Tung VT, Gravningen K. 2010. Experimental challenge studies in Vietnamese catfish, *Pangasianodon hypophthalmus* (Sauvage), exposed to *Edwardsiella ictaluri* and *Aeromonas hydrophila*. J Fish Dis 33:717–722. doi:10.1111/j.1365-2761.2010.01173.x. 20572902

[22] Hoang AH, Tran TTX, Le PN, Dang THO. 2019. Selection of phages to control *Aeromonas hydrophila*—an infectious agent in striped catfish. Biocontrol Sci 24:23–28. doi:10.4265/bio.24.23. 30880310

[23] Baumgartner WA, Ford L, Hanson L. 2017. Lesions caused by virulent *Aeromonas hydrophila* in farmed catfish (*Ictalurus punctatus* and *I. punctatus* × *I. furcatus*) in Mississippi. J Vet Diagn Invest 29:747–751. doi:10.1177/1040638717708584. 28482758

[24] Nahar S, Rahman M, Ahmed G, Faruk M. 2016. Isolation, identification, and characterization of *Aeromonas hydrophila* from juvenile farmed pangasius (*Pangasianodon hypophthalmus*). Int J Fish Aquat 4:52–60.

[25] Zhou T, Yuan Z, Tan S, Jin Y, Yang Y, Shi H, Wang W, Niu D, Gao L, Jiang W, Gao D, Liu Z. 2018. A review of molecular responses of catfish to bacterial diseases and abiotic stresses. Front Physiol 9:1113. doi:10.3389/fphys.2018.01113. 30210354 PMC6119772

[26] Li C, Wang R, Su B, Luo Y, Terhune J, Beck B, Peatman E. 2013. Evasion of mucosal defenses during *Aeromonas hydrophila* infection of channel catfish (*Ictalurus punctatus*) skin. Dev Comp Immunol 39:447–455. doi:10.1016/j.dci.2012.11.009. 23219904

[27] Li C, Beck B, Su B, Terhune J, Peatman E. 2013. Early mucosal responses in blue catfish (*Ictalurus furcatus*) skin to *Aeromonas hydrophila* infection. Fish Shellfish Immunol 34:920–928. doi:10.1016/j.fsi.2013.01.002. 23337110

[28] Siao R-F, Lin C-H, Chen L-H, Wang L-C. 2021. Establishment of a striped catfish skin explant model for studying the skin response in *Aeromonas hydrophila* infections. Sci Rep 11:19057. doi:10.1038/s41598-021-98583-8. 34561532 PMC8463585

[29] Lokesh J, Kiron V. 2016. Transition from freshwater to seawater reshapes the skin-associated microbiota of Atlantic salmon. Sci Rep 6:19707. doi:10.1038/srep19707. 26806545 PMC4726331

[30] Ben Hamed S, Guardiola F, Mars M, Esteban MA. 2014. Pathogen bacteria adhesion to skin mucus of fishes. Vet Microbiol 171:1–12. doi:10.1016/j.vetmic.2014.03.008. 24709124

[31] Balasubramanian S, Prakash M, Senthilraja P, Gunasekaran G. 2012. Antimicrobial properties of skin mucus from four freshwater cultivable fishes (*Catla catla*, *Hypophthalmichthys molitrix*, *Labeo rohita* and *Ctenopharyngodon idella*). Afr J Microbiol Res 6:5110–5120.

[32] Kumari U, Nigam AK, Mitial S, Mitial AK. 2011. Antibacterial properties of the skin mucus of the freshwater fishes, *Rita rita* and *Channa punctatus*. Eur Rev Med Pharmacol Sci 15:781–786. 21780547

[33] Austin B. 2002. The bacterial microflora of fish. ScientificWorldJournal 2:558–572. doi:10.1100/tsw.2002.137. 12805983 PMC6009360

[34] Cao H, He S, Wei R, Diong M, Lu L. 2011. *Bacillus amyloliquefaciens* G1: a potential antagonistic bacterium against eel-pathogenic *Aeromonas hydrophila*. Evid Based Complement Alternat Med 2011:824104. doi:10.1155/2011/824104. 21754944 PMC3132486

[35] Hoque F, Jawahar Abraham T, Nagesh TS, Kamilya D. 2019. *Pseudomonas aeruginosa* FARP(72) offers protection against *Aeromonas hydrophila* infection in *Labeo rohita*. Probiotics Antimicrob Proteins 11:973–980. doi:10.1007/s12602-018-9456-1. 30112591

[36] Koch BEV, Yang S, Lamers G, Stougaard J, Spaink HP. 2018. Intestinal microbiome adjusts the innate immune setpoint during colonization through negative regulation of MyD88. Nat Commun 9:4099. doi:10.1038/s41467-018-06658-4. 30291253 PMC6173721

[37] Murdoch CC, Rawls JF. 2019. Commensal microbiota regulate vertebrate innate immunity—insights from the zebrafish. Front Immunol 10:2100. doi:10.3389/fimmu.2019.02100. 31555292 PMC6742977

[38] Rolig AS, Parthasarathy R, Burns AR, Bohannan BJ, Guillemin K. 2015. Individual members of the microbiota disproportionately modulate host innate immune responses. Cell Host Microbe 18:613–620. doi:10.1016/j.chom.2015.10.009. 26567512 PMC4701053

[39] Pérez T, Balcázar JL, Ruiz-Zarzuela I, Halaihel N, Vendrell D, de Blas I, Múzquiz JL. 2010. Host-microbiota interactions within the fish intestinal ecosystem. Mucosal Immunol 3:355–360. doi:10.1038/mi.2010.12. 20237466

[40] Tarnecki A, Wafapoor M, Phillips R, Rhody N. 2019. Benefits of a Bacillus probiotic to larval fish survival and transport stress resistance. Sci Rep 9:4892. doi:10.1038/s41598-019-39316-w. 30894554 PMC6426941

[41] Cámara-Ruiz M, Balebona MC, Moriñigo MÁ, Esteban MÁ. 2020. Probiotic *Shewanella putrefaciens* (SpPdp11) as a fish health modulator: a review. Microorganisms 8:1990. doi:10.3390/microorganisms8121990. PMC7764857 33327443

[42] Qin C, Xie Y, Wang Y, Li S, Ran C, He S, Zhou Z. 2018. Impact of *Lactobacillus casei* BL23 on the host transcriptome, growth and disease resistance in larval zebrafish. Front Physiol 9:1245. doi:10.3389/fphys.2018.01245. 30233415 PMC6131626

[43] de Souza Valente C, Wan A. 2021. *Vibrio* and major commercially important vibriosis diseases in decapod crustaceans. J Invertebr Pathol 181:107527. doi:10.1016/j.jip.2020.107527. 33406397

[44] Chatterjee S. 2012. *Vibrio* related diseases in aquaculture and development of rapid and accurate identification methods. J Mar Sci Res Dev S1:e002.

[45] Li J, Woo N. 2003. Pathogenicity of vibrios in fish: an overview. J Ocean Univ China 2:117–128. doi:10.1007/s11802-003-0039-7.

[46] Zhang X, Ding L, Yu Y, Kong W, Yin Y, Huang Z, Zhang X, Xu Z. 2018. The change of teleost skin commensal microbiota is associated with skin mucosal transcriptomic responses during parasitic infection by *Ichthyophthirius multifillis*. Front Immunol 9:2972. doi:10.3389/fimmu.2018.02972. 30619329 PMC6305302

[47] Boutin S, Bernatchez L, Audet C, Derôme N. 2013. Network analysis highlights complex interactions between pathogen, host and commensal microbiota. PLoS One 8:e84772. doi:10.1371/journal.pone.0084772. 24376845 PMC3871659

[48] Baya A, Lupiani B, Bandin I, Hetrick FM, Figueras A, Carnanan A, May EM, Toranzo A. 1992. Phenotypic and pathobiological properties of Corynebacterium aquaticum isolated from diseased striped bass. Dis Aquat Org 14:115–126. doi:10.3354/dao014115.

[49] Dong S, Ding L-G, Cao J-F, Liu X, Xu H-Y, Meng K-F, Yu Y-Y, Wang Q, Xu Z. 2019. Viral-infected change of the digestive tract microbiota associated with mucosal immunity in teleost fish. Front Immunol 10:2878. doi:10.3389/fimmu.2019.02878. 31921142 PMC6930168

[50] Ryan MP, Pembroke JT. 2018. Brevundimonas spp: emerging global opportunistic pathogens. Virulence 9:480–493. doi:10.1080/21505594.2017.1419116. 29484917 PMC5955483

[51] Ray D, Kidane D. 2016. Gut microbiota imbalance and base excision repair dynamics in colon cancer. J Cancer 7:1421–1430. doi:10.7150/jca.15480. 27471558 PMC4964126

[52] Elias PM, Brown BE, Ziboh VA. 1980. The permeability barrier in essential fatty acid deficiency: evidence for a direct role for linoleic acid in barrier function. J Invest Dermatol 74:230–233. doi:10.1111/1523-1747.ep12541775. 7373078

[53] Fjelldal P, Hansen T, Karlsen Ø. 2020. Effects of laboratory salmon louse infection on osmoregulation, growth and survival in Atlantic salmon. Conserv Physiol 8:coaa023. doi:10.1093/conphys/coaa023. 32257215 PMC7098368

[54] Al-Harbi A, Uddin M. 2004. Seasonal variation in the intestinal bacterial flora of hybrid tilapia (*Oreochromis niloticus* × *Oreochromis aureus*) cultured in earthen ponds in Saudi Arabia. Aquaculture 229:37–44. doi:10.1016/S0044-8486(03)00388-0.

[55] Llewellyn MS, Boutin S, Hoseinifar SH, Derome N. 2014. Teleost microbiomes: the state of the art in their characterization, manipulation and importance in aquaculture and fisheries. Front Microbiol 5:207. doi:10.3389/fmicb.2014.00207. 24917852 PMC4040438

[56] Lowrey L, Woodhams DC, Tacchi L, Salinas I. 2015. Topographical mapping of the rainbow trout (*Oncorhynchus mykiss*) microbiome reveals a diverse bacterial community with antifungal properties in the skin. Appl Environ Microbiol 81:6915–6925. doi:10.1128/AEM.01826-15. 26209676 PMC4561705

[57] Goto Y. 2019. Epithelial cells as a transmitter of signals from commensal bacteria and host immune cells. Front Immunol 10:2057. doi:10.3389/fimmu.2019.02057. 31555282 PMC6724641

[58] American Veterinary Medical Association. 2020. AVMA guidelines for the euthanasia of animals. American Veterinary Medical Association, Schaumburg, IL. https://www.avma.org/resources-tools/avma-policies/avma-guidelines-euthanasia-animals.

[59] Tang C-H, Lai D-Y, Lee T-H. 2012. Effects of salinity acclimation on Na+/K+-ATPase responses and FXYD11 expression in the gills and kidneys of the Japanese eel (*Anguilla japonica*). Comp Biochem Physiol A Mol Integr Physiol 163:302–310. doi:10.1016/j.cbpa.2012.07.017. 22885345

[60] Livak KJ, Schmittgen TD. 2001. Analysis of relative gene expression data using real-time quantitative PCR and the 2−ΔΔCT method. Methods 25:402–408. doi:10.1006/meth.2001.1262. 11846609

[61] Quast C, Pruesse E, Yilmaz P, Gerken J, Schweer T, Yarza P, Peplies J, Glöckner FO. 2013. The SILVA ribosomal RNA gene database project: improved data processing and Web-based tools. Nucleic Acids Res 41:D590–D596. doi:10.1093/nar/gks1219. 23193283 PMC3531112

[62] Oksanen J, Blanchet FG, Kindt R, Legendre P, Minchin P, O’Hara B, Simpson G, Solymos P, Stevens H, Wagner H. 2015. Vegan: community ecology package. R package version 22-1 2:1-2.

[63] Chong J, Liu P, Zhou G, Xia J. 2020. Using MicrobiomeAnalyst for comprehensive statistical, functional, and meta-analysis of microbiome data. Nat Protoc 15:799–821. doi:10.1038/s41596-019-0264-1. 31942082

[64] Dhariwal A, Chong J, Habib S, King IL, Agellon LB, Xia J. 2017. MicrobiomeAnalyst: a Web-based tool for comprehensive statistical, visual and meta-analysis of microbiome data. Nucleic Acids Res 45:W180–W188. doi:10.1093/nar/gkx295. 28449106 PMC5570177

[65] Liu P-Y, Cheng A-C, Huang S-W, Lu H-P, Oshida T, Liu W, Yu H-T. 2020. Body-size scaling is related to gut microbial diversity, metabolism and dietary niche of arboreal folivorous flying squirrels. Sci Rep 10:7809. doi:10.1038/s41598-020-64801-y. 32385374 PMC7210948

