# Supplementary figures and images for: Aeromonas hydrophila Induces Skin Disturbance through Mucosal Microbiota Dysbiosis in Striped Catfish (Pangasianodon hypophthalmus)
Source: mSphere. 2022 Jun 29;7(4):e00194-22. doi: 10.1128/msphere.00194-22 (PMC9429897; doi:10.1128/msphere.00194-22)

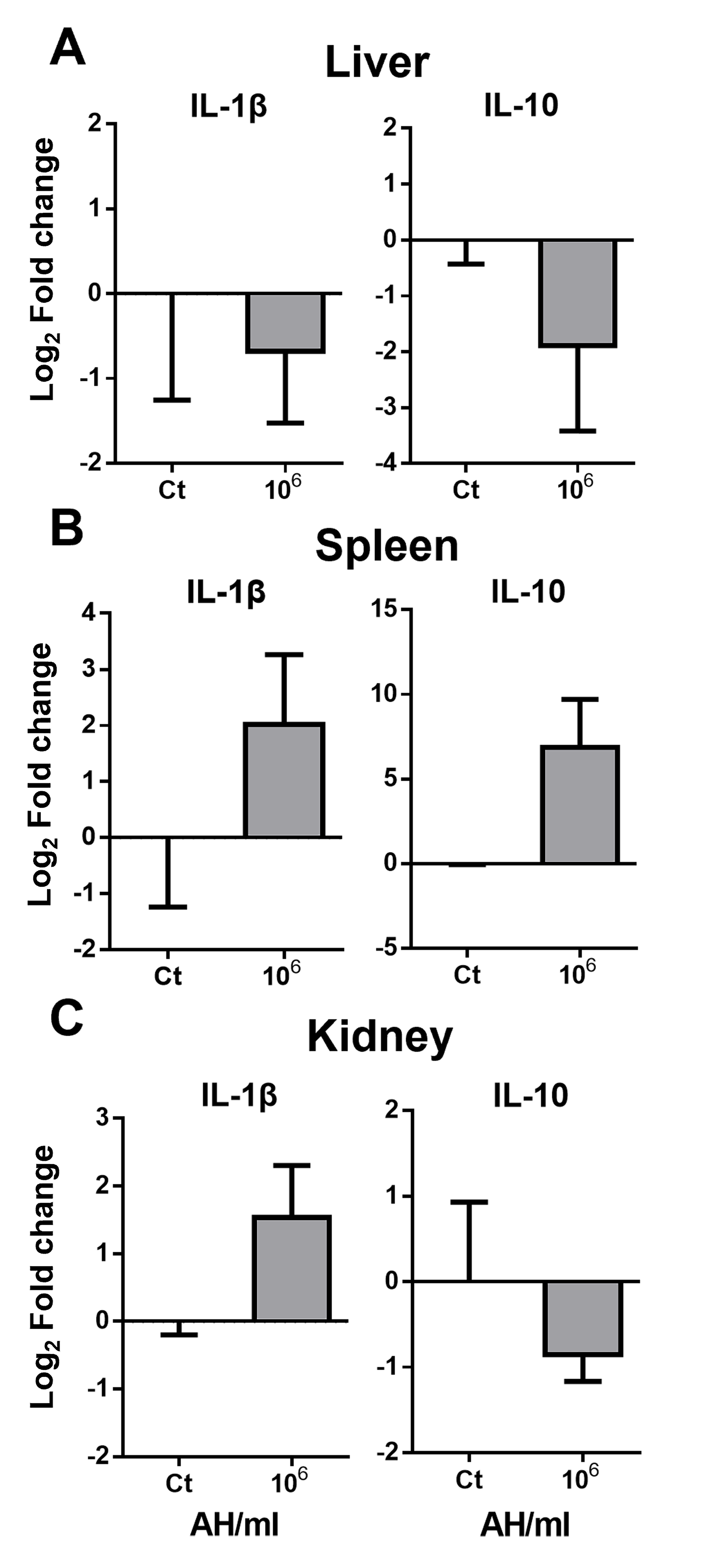

Supplement: FIG S1 [file msphere.00194-22-s0005.tif]

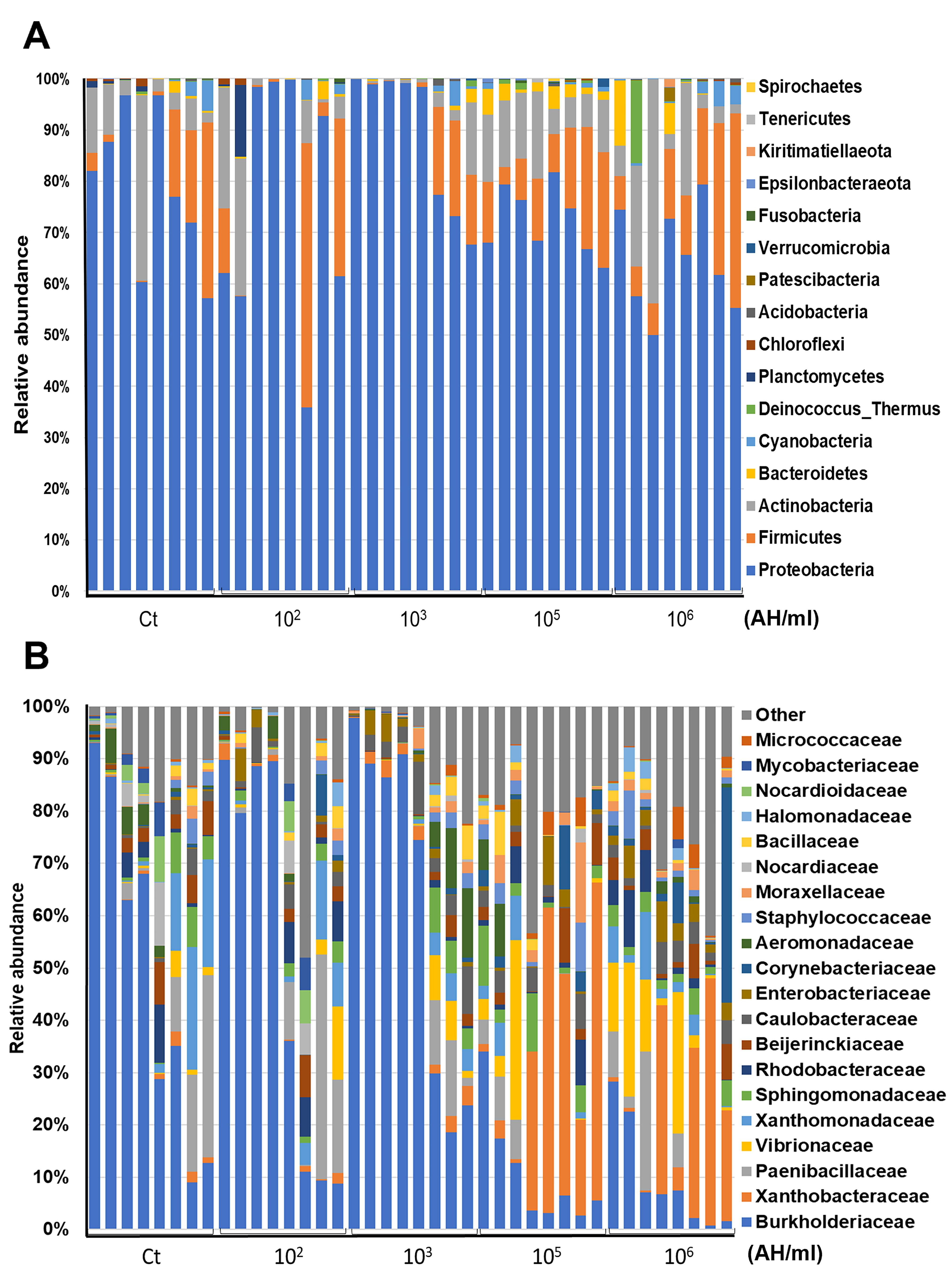

Supplement: FIG S2 [file msphere.00194-22-s0006.jpg]

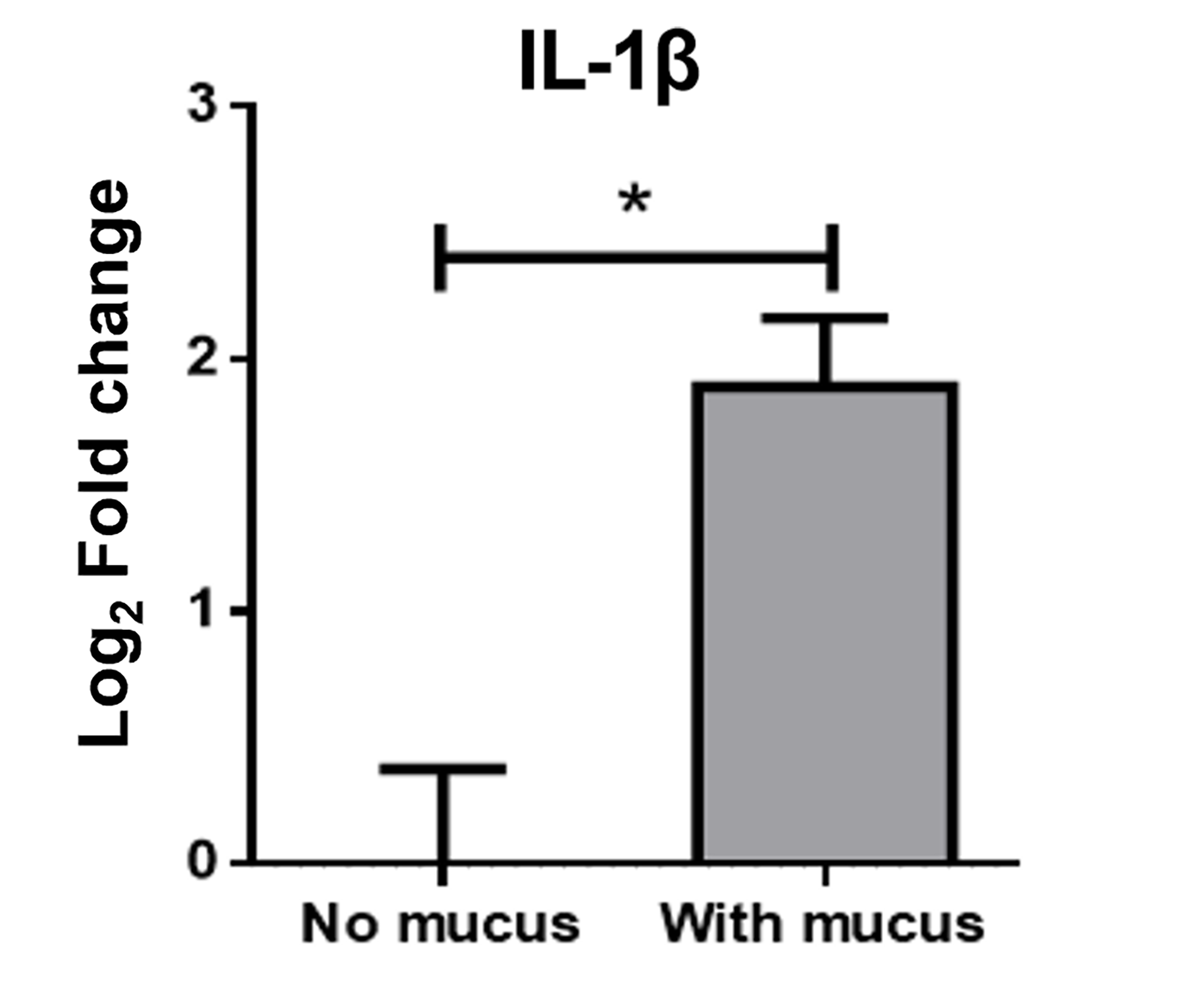

Supplement: FIG S3 [file msphere.00194-22-s0007.tif]

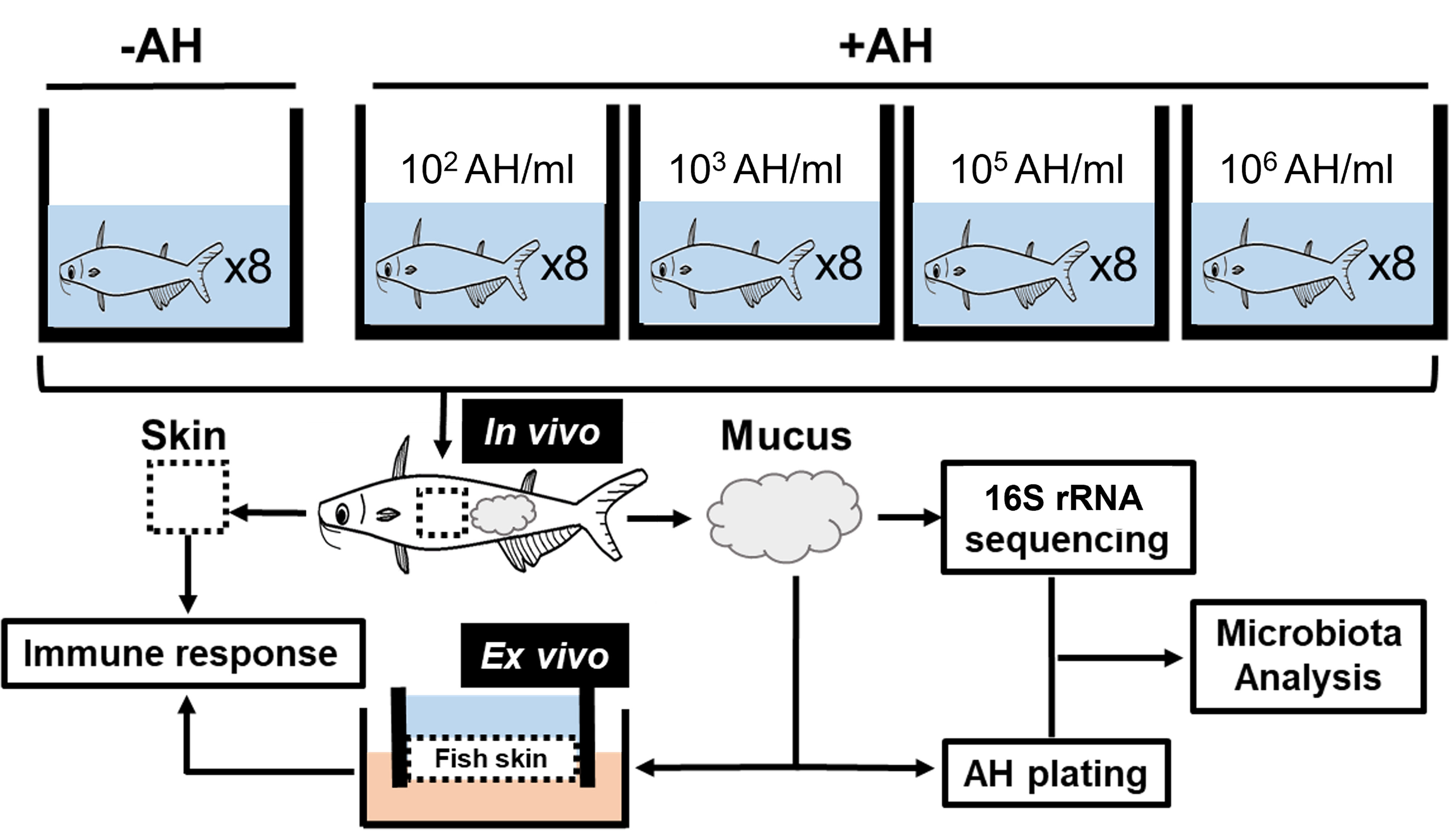

Supplement: FIG S4 [file msphere.00194-22-s0008.jpg]

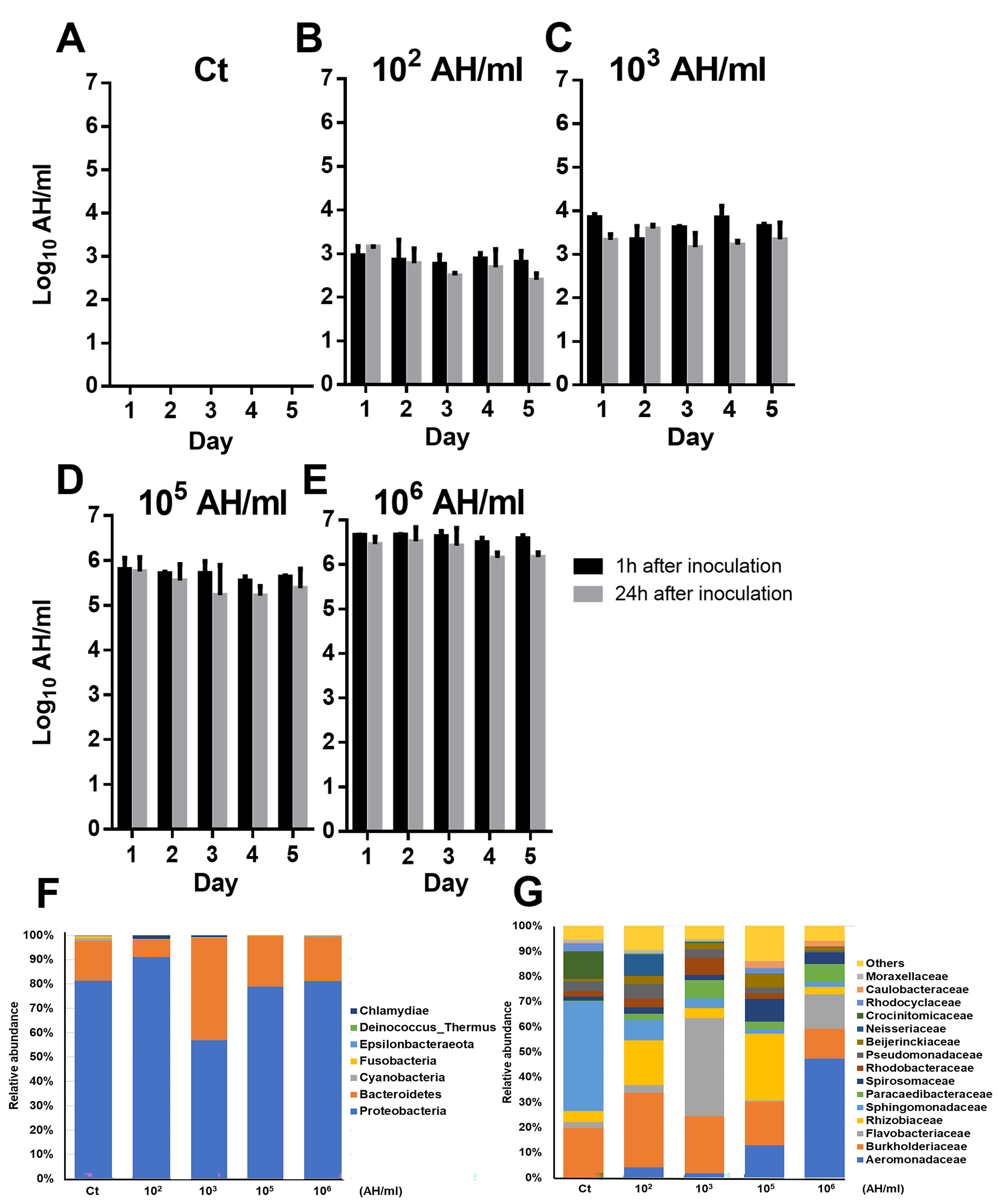

Supplement: FIG S5 [file msphere.00194-22-s0009.jpg]
