# Supplementary material for: Aeromonas hydrophila Induces Skin Disturbance through Mucosal Microbiota Dysbiosis in Striped Catfish (Pangasianodon hypophthalmus)
Source: mSphere. 2022 Jun 29;7(4):e00194-22. doi: 10.1128/msphere.00194-22 (PMC9429897; doi:10.1128/msphere.00194-22)
Supplement: TABLE S1 [file msphere.00194-22-s0001.docx]

**TABLE S1 AH was examined in liver, spleen and posterior kidney in both control and 10^6^ AH/ml challenge group.**

|  | Liver | | | | |  | Spleen | | | | |  | Kidney | | | | |
| --- | --- | --- | --- | --- | --- | --- | --- | --- | --- | --- | --- | --- | --- | --- | --- | --- | --- |
| Group/Fish number | 1 | 2 | 3 | 4 | 5 |  | 1 | 2 | 3 | 4 | 5 |  | 1 | 2 | 3 | 4 | 5 |
| Ct | **-** | **-** | **-** | **-** | **-** |  | **-** | **-** | **-** | **-** | **-** |  | **-** | **-** | **-** | **-** | **-** |
| 10^6^AH/ml | **-** | **-** | **-** | **-** | **-** |  | **-** | **-** | **-** | **-** | **-** |  | **-** | **-** | **-** | **-** | **-** |
