# Supplementary material for: Aeromonas hydrophila Induces Skin Disturbance through Mucosal Microbiota Dysbiosis in Striped Catfish (Pangasianodon hypophthalmus)
Source: mSphere. 2022 Jun 29;7(4):e00194-22. doi: 10.1128/msphere.00194-22 (PMC9429897; doi:10.1128/msphere.00194-22)
Supplement: TABLE S2 [file msphere.00194-22-s0002.docx]

**TABLE S2 Statistical analysis was applied to determine the significant difference of relative abundance of bacteria genera between each AH-challenged group to non-AH control. (One-way ANOVA followed by Dunnett's post-hoc test, ****P* ≤ 0.001; ***P* ≤ 0.01; **P* ≤ 0.05, NS for no significance)**

| Relative abundance of genera | Ct vs 10^2^ | Ct vs 10^3^ | Ct vs 10^5^ | Ct vs 10^6^ |
| --- | --- | --- | --- | --- |
| *Vibrio* | NS | NS | NS | * |
| *Corynebacterium_1* | NS | NS | NS | NS |
| *Paracoccus* | NS | NS | NS | NS |
| *Brevundimonas* | NS | NS | NS | NS |
| *Escherichia_Shigella* | NS | NS | NS | * |
