# Supplementary material for: Aeromonas hydrophila Induces Skin Disturbance through Mucosal Microbiota Dysbiosis in Striped Catfish (Pangasianodon hypophthalmus)
Source: mSphere. 2022 Jun 29;7(4):e00194-22. doi: 10.1128/msphere.00194-22 (PMC9429897; doi:10.1128/msphere.00194-22)
Supplement: TABLE S3 [file msphere.00194-22-s0003.docx]

**TABLE S3 Anti-AH property of the skin mucus was examined by different treatments included the positive control of tetracycline, the negative control of double-distilled water and the different concentration of mucus.**

|  |  |  | Treatment |  |  |
| --- | --- | --- | --- | --- | --- |
| *Aeromonas hydrophila*  concentration | tetracycline  (50mg/μl) | double-distilled  water | mucus  (1X) | mucus  (1X/10) | mucus  (1X/100) |
| 10^5^AH/ml | **+** | **-** | **-** | **-** | **-** |
| 10^6^AH/ml | **+** | **-** | **-** | **-** | **-** |
| 10^7^AH/ml | **+** | **-** | **-** | **-** | **-** |
