# Supplementary material for: Aeromonas hydrophila Induces Skin Disturbance through Mucosal Microbiota Dysbiosis in Striped Catfish (Pangasianodon hypophthalmus)
Source: mSphere. 2022 Jun 29;7(4):e00194-22. doi: 10.1128/msphere.00194-22 (PMC9429897; doi:10.1128/msphere.00194-22)
Supplement: TEXT S1 [file msphere.00194-22-s0010.docx]

**TEXT S1 Supplemental Materials and Methods**

***Ex vivo* fish skin model system**

A striped catfish model system based on was applied in this research. The striped catfish skin tissue was removed from the fish by scalpel and immediately immersed into cold L-15 medium (SIGMA, USA) supplemented with 10% fetal bovine serum, 2% gentamycin solution (SIGMA, USA), 1X antibiotic-antimycotic (Biowest, USA). After cutting into squares of approximately 10×10 mm, skin tissue was fixed in the upper plastic crown using a fine rubber band and mounted with the lower plastic crown. The tissue and the crown were gently submerged in the culture medium in a 24-well culture plate. The plate was cultured in a CO_2_-free incubator at 25°C. The culture medium was changed every two days until further experiments were conducted. The media was replaced with non-antibiotic media 24 hr before the infection experiment.

**Total RNA extraction and cDNA preparation**

Each total RNA sample from the skin, liver, spleen, and kidney of striped catfish were extracted with the TriPure Isolation Reagent (Roche, Mannheim, Germany) following the manufacturer's instructions. Extracted RNA samples were dissolved in 30 μl of sterilized DDW and stored at −80 °C after isolation. The concentration and quality of extracted RNA were measured by DS-11 Series Spectrophotometer (DeNovix, USA). Purified RNA with an A260/A280 ratio between 1.8 and 2.0 was used for all RNA experiments. First-strand cDNA was synthesized from 2 mg of total RNA by M-MLV Reverse transcriptase (Promega, USA) following the manufacturer's instructions. The successful construction of cDNA library was determined by 1.5% agarose gels containing the Safeview DNA stain (GeneMark, Taiwan).

**Real-Time PCR (qPCR)**

Primer pairs were designed using Universal Probe library website and shown in Table S4. All the cDNA products were utilized for the qPCR reaction using the GoTaq qPCR Master Mix on a CFX96 real-time PCR Detection System (Bio-Rad, CA). The thermal cycling profile consisted of an initial denaturation at 95^o^C (for 2 min), followed by 40 cycles of denaturation at 95^o^C (3 sec), an appropriate annealing/extension temperature (60^o^C, 30 sec). An additional temperature ramping step was utilized to produce melting curves of the reaction from 65^o^C to 95^o^C. The housekeeping gene EF1-α was set as the reference gene, relative fold changes were calculated based on the cycle threshold (Ct) values generated by qPCR. Expression differences between control and treatment groups were assessed for statistical significance using a randomization test in the GraphPad Prism. The numbers in y-axes correspond to the Log_2_ fold increase compared to the non-AH challenge group.

**Skin mucus microbial DNA extraction**

*P. hypophthalmus* skin mucus about 20 μl was homogenized in 100 μl of TE buffer (1 M, pH 8 Tris-Cl; 0.5M, pH 8 EDTA) and 100 μl lysozyme (50 mg/ml) then incubated at 37°C for 30 min. Then, TE buffer, 10% SDS, and proteinase K (20 μg/ml) were added, and the sample was incubated at 56°C for 1 hr. After that, 5 M NaCl and phenol:chloroform:isoamyl alcohol (25:24:1) was added to the sample and centrifuged. chloroform:isoamyl (24:1) was added to the collected supernatant and was also centrifuged. The DNA was precipitated with 90% ethanol, put in -20°C overnight, and centrifuged the next day again. The DNA pellet was rinsed with 70% ethanol and centrifuged once more, air-dried in a laminar flow hood, and resuspended in TE buffer or double-distilled water. All centrifugation steps were performed at 14000 rpm, 5 min, and 4°C.

**16S rRNA gene sequencing and community analyses**

Skin mucus microbial DNA was extracted and assessed photometrically using DS-11 Series Spectrophotometer (DeNovix, USA). The V3-V4 hypervariable region of 16S rRNA genes was amplified by the primer set 341F (5′-CCTACGGGNGGCWGCAG-3′) and 805R (5′-GACTACHVGGGTATCTAATCC-3′). After preparation and quality control, the 16S rRNA libraries were sequenced on the Illumina MiSeq platform system. After sequencing, we obtained 50000-100000 raw reads from the original samples. The software system Quantitative Insights Into Microbial Ecology (QIIME2.2019.10) was used to conduct the quality filter of raw data. The DADA2 method for ASV inference was used to process the 16S rRNA gene amplicon data. Silva database classifier was applied for the taxonomic assignment. Microbial community analyses were conducted with Rstudio (R version 4.1.1) package vegan by referenced scripts and MicrobiomeAnalyst. Alpha diversity was analyzed based on the ASV table and was analyzed by Rstudio, followed by a one-way ANOVA and post-hoc Tukey HSD comparisons under *P < 0.05*. For Beta diversity, dissimilarities among microbial communities were measured by Bray-Curtis distance and conducted with principal coordinates analysis (PCoA) based on the bacterial classification of ASVs and was drawn by Rstudio. In further examination, Adonis pairwise comparisons of microbiota composition were applied based on bray-Curtix distances under *P* < 0.05 for all the statistical analyses between each group. Different letters shown on the top of the boxplot in Alpha and Beta diversity boxplot indicated the differences under statistical analyses.

LEfSe was applied for identifying featured mucus microbes among striped catfish by using relative abundances of ASVs. The featured ASVs were first tested and detected with a Kruskal-Wallis test (*P-value* cutoff=0.1); then, linear discriminant analysis (LDA) was conducted for selecting featured ASVs by effect size (absolute value of the logarithmic LDA score=2).

**Functions prediction of microbial community**

PICRUSt2 was used to predict the metagenome to evaluate potential functions of striped catfish’ skin microbiota. Copy number adjusted ASV tables were used for predicting Kyoto Encyclopedia of Genes and Genomes (KEGG) Orthology (KO) abundances. The KOs were categorized by KEGG pathway database and specific metabolic module/reactions of interest. Pathway/module topology analysis was conducted with the Rstudio (R version 4.1.1) package path view by referenced scripts.
