# Supplementary material for: Aeromonas hydrophila Induces Skin Disturbance through Mucosal Microbiota Dysbiosis in Striped Catfish (Pangasianodon hypophthalmus)
Source: mSphere. 2022 Jun 29;7(4):e00194-22. doi: 10.1128/msphere.00194-22 (PMC9429897; doi:10.1128/msphere.00194-22)
Supplement: TABLE S4 [file msphere.00194-22-s0004.docx]

**TABLE S4 Primer pairs used in this research.**

| Primer pair | Sequence (5’-3’) | Target Gene |
| --- | --- | --- |
| EF1A F  EF1A R | AGGACATCCGTCGTGGTAAC  TCAGGATGATGACCTGAGCA | Elongation factor 1-alpha |
| MyD88 F  MyD88 R | TGTTTGACCGTGACGTCCT  TCTTGCACCTTCTCTCGATG | Myeloid differentiation primary response 88 |
| IL-1β F  IL-1β R | CAGTGCAAATGTGTCAGCAG  GGTCTCATCATGAAGCGTGA | Interleukin 1 beta |
| TLR4 F  TLR4 R | GCAAGGCAATGCTAACATCAT  GAAGACCAAACACCTTCTTTGAC | Toll-like receptor 4 |
| TLR5 F  TLR5 R | TCATCTCGGTTCTGTTGATCC  AAGCGGTTTCCAGACAACTC | Toll-like receptor 5 |
| Muc5AC F  Muc5AC R | GTGTTTCAATACTACGACACGG  ACAAAGGGCAACACGTTCAC | Mucin-5AC |
| IL-8 F  IL-8 R | CCC CTC AGC TCT AGC TGT TAC T  CGG GGC TGT ATT AGA AGT CCT | Interleukin 8 |
| IL-10 F  IL-10 R | CGC CAA GAT CCG AGA CTA CT  CAT CCG TAA GGG CTT TTG AA | Interleukin 10 |
